# Gut Microbiome Alterations in TAAR5 Knockout Mice and In Silico Ligand Prediction

**DOI:** 10.3390/ijms27198493

**Published:** 2026-09-23

**Authors:** Ilya S. Zhukov, Yazen Alnefeesi, Nikita S. Gladyshev, Alexey Zolkin, Alena B. Zalicheva, Yashar Chalabiani, Ildikó Miklya, Elena I. Ermolenko, Raul R. Gainetdinov

**Affiliations:** 1 Institute of Translational Biomedicine, St. Petersburg State University, 199034 St. Petersburg, Russia; 2 Institute of Experimental Medicine, 197376 St. Petersburg, Russia; 3 Avtsyn Research Institute of Human Morphology, Petrovsky National Research Centre of Surgery, 119991 Moscow, Russia; 4 Department of Pharmacology and Pharmacotherapy, Semmelweis University, 1085 Budapest, Hungary; 5 Center for Pharmacology and Drug Research & Development, Semmelweis University, 1085 Budapest, Hungary

**Keywords:** gut microbiota, TAAR5, knockout mice, *Ruminococcus*, in silico docking, microbiome, trimethylamine, host-microbe interaction, 16S rRNA sequencing, GPCR receptor

## Abstract

Trace amine-associated receptors (TAARs) are a class of G protein-coupled receptors with roles in olfaction, behaviour, and immunity. Known best as an olfactory receptor, TAAR5 has been linked to emotional behaviour and hippocampal neurogenesis, yet its peripheral mechanisms remain poorly understood. Given bidirectional influences between the gut microbiota and the host, we investigated whether TAAR5 deletion alters the gut microbiome in mice. Using 16S rRNA sequencing, we compared the gut microbiota of TAAR5-knockout (TAAR5-KO) and wild-type mice. While alpha-diversity remained unchanged, beta-diversity analysis revealed significant separation between genotypes, with TAAR5-KO mice exhibiting markedly increased inter-individual variability. Taxonomically, KO mice showed a significant reduction in *Ruminococcus* and ASF356, alongside an enrichment of specific *Lachnospiraceae* and *Prevotellaceae* lineages. Functional metagenomic prediction (PICRUSt2) implied a reduction in the metagenomic capacity for central metabolic pathways (glycolysis, TCA cycle, fatty acid β-oxidation) and an increase in the capacity for guanosine nucleotide degradation pathways; these hypotheses remain untested. Molecular docking identified candidate microbial metabolites, including small amines (2-aminophenol, aminoacetone, butan-1-amine), with ligand efficiencies comparable to the known TAAR5 agonist trimethylamine. These findings suggest that TAAR5 may influence gut microbiome composition and function, with particular impact on *Ruminococcus*—a genus linked to intestinal inflammation. The increased compositional variability in TAAR5-KO mice suggests that TAAR5 may act as a homeostatic gatekeeper in gut microbial ecology. Our in silico results offer testable hypotheses for future validation of microbial metabolites as novel TAAR5 ligands.

## 1. Introduction

Trace amines (TAs) and their family of GPCR receptors (TAARs) represent a unique neurotransmitter system with significant pharmacological potential [1]. For decades, research has predominantly focused on the chemosensory functions of TAARs within the central nervous system (CNS). However, emerging evidence suggests that their peripheral actions may exert a more profound influence on overall physiology than previously appreciated. The TAARs have undergone three, mostly separate lines of study since their discovery at the turn of the century. Fraught with striking interspecies differences [2,3,4,5], these lines of evidence arrive at distinct characterizations of the TAAR family: (1) the TAARs are olfactory receptors expressed in the intranasal epithelium [6,7,8], (2) the TAARs directly regulate brain functions outside the olfactory epithelium [9,10,11,12], and (3) the TAARs regulate peripheral functions, immunity, and cancer [13,14,15,16,17,18,19,20]. All human TAARs (TAAR1, TAAR2, TAAR5, TAAR6, TAAR8, and TAAR9) represent promising targets for drug development aimed at both CNS and peripheral disorders [21]. In both primates and rodents, the TAAR genes are distributed consecutively in a single locus, and the number of gene duplications varies widely across species [2,3,4]. This hints at the possibility that these genes have taken on different functions in different species.

The gut microbiome communicates with the host via a “chemical language” that includes microbially derived TAs and other neuroactive molecules [22]. The gastrointestinal tract represents a primary site for high concentrations of TAs, positioning intestinal TAARs as potential key sensors of microbial metabolism [23]. This is supported by their ability to be activated by microbially derived amines, such as trimethylamine (TMA) for TAAR5 [24], and by the expression of multiple TAARs, including TAAR1, TAAR9, and TAAR5, in the gut epithelium [25]. In the brain, a transgenic TAAR5-KO and LacZ reporter line shows staining as deep as the dentate gyrus [26], where increased neurogenesis was confirmed in these mutants [11]. Recently, TAAR5 activation by the gut microbial metabolite, trimethylamine (TMA), was found to increase inflammatory and fibrotic markers in primary cultures of human intestinal myofibroblasts [27], expanding the clinical relevance of TAAR5 to irritable bowel syndrome (IBD) and Crohn’s disease, beyond psychiatry. Primary cultures of human immune cells also express TAAR5 [17], and many such immune cells are known to patrol the gut and shape the composition of the microbiota [28,29,30,31].

One of the known mechanisms of immune control over the composition of the gut microbiota relies on immune responses to antigens detected at the basal side of the gut mucosa. Ultimately, B-cells secrete microbe-specific antibodies at the basal membrane of the gut epithelium [28,32], and are transported by various means into the gut lumen, where they engulf the target microbe [30], to curb the expansion of its population. The study of such pathways has generated a wealth of data mapping the interface between secretory antibodies and the gut microbiota [33]. Importantly, human B-cells and several other immune cells appreciably express both TAAR5 and TAAR9 [17]. We had also found previously that ablation of *Taar1* and *Taar9* alters the gut microbiota in rodents [34,35]. Furthermore, the only known endogenous agonist of TAAR5 is TMA, and its only known endogenous source is the gut flora [24]. We thus tested whether knockout of TAAR5 (TAAR5-KO) in mice alters the composition of the gut microbiota.

Despite its pharmacological potential, TAAR5 remains a challenging target for drug discovery, with few ligands characterized and a clear need for specific modulators [36]. In the present study, we investigated the impact of TAAR5-KO on the gut microbiota using 16S rRNA sequencing, functional metagenomic predictions, and in silico docking to explore the chemical space of microbial metabolites and identify candidate ligands.

## 2. Results

### 2.1. Gut Microbial Composition Varies Widely in TAAR5-KO but Not WT Mice

Alpha-diversity analysis revealed no significant differences in richness or evenness between WT and KO mice as assessed by both traditional indices (Shannon, Simpson, Pielou) and Hill numbers (q = 0, 1, 2) (Figure 1a). Beta-diversity analysis using Bray–Curtis dissimilarities showed a clear separation by genotype. Concretely, pairwise dissimilarities within the KO group were significantly larger than those within the WT group, rivalling inter-group dissimilarities in terms of variance (Figure 1b). Hierarchical clustering by this dissimilarity matrix produced a cluster of *n* = 4 KO mice, a larger cluster including all WT and two KO mice, and yet another pair of outlying KO samples (Figure 1c). Principal Coordinate Analysis (PCoA) recapitulated the greater dispersion of the KO group, whose centroid had significantly shifted from the more compact WT cluster (PERMANOVA, *p* < 0.01). The first two principal coordinates (Figure 1d) explained 39.5% of the total variance in pairwise Bray–Curtis dissimilarities and adequately captured the clustering pattern revealed in Figure 1c. Given a KO sample of *n* = 8, the mutant subgroups were too small to substantiate by statistical analysis, but the PCoA plot (Figure 1d) shows qualitative differences. To confirm that the observed separation was not driven solely by abundant lineages, we additionally calculated generalized UniFrac distances (α = 0.5). PERMANOVA on generalized UniFrac distances confirmed a significant genotype effect (R^2^ = 0.158, F = 3.38, *p* = 0.0051), and PERMDISP revealed significantly greater multivariate dispersion in the KO group (F = 8.03, *p* = 0.011), indicating that TAAR5-KO mice exhibited higher inter-individual variability at the phylogenetic level (Supplementary Figure S4).

### 2.2. Deletion of TAAR5 Depletes Ruminococcus *spp.* and Others in the Gut

Interestingly, a subset of KO samples diverged along the PCoA axes, which correlated with specific taxon losses: separation along the first coordinate was associated with a decrease in *Prevotellaceae* and an increase in Incertae Sedis, while divergence along the second coordinate corresponded with a marked loss of *Turicimonas* (Figure 2a); but there are important caveats here. The effect spanning PC1 explained ~24% of the total variance (outliers S7 and S6 of note), and there is a *p* = 0.18 of observing a similar or more extreme effect on KO mice if this effect happens randomly to mice at *p* = 0.1. Therefore, we cannot state that the substitution of *Prevotellaceae* with Incertae Sedis is linked to TAAR5-KO. The *Turicimonas* effect on PC2 applied to 5/8 KO samples and to none of the 12 controls, which gives *p* = 0.03 for observing a similar or more extreme effect, with *p* = 0.25 being the probability of a random effect. This links the *Turicimonas* reduction along PC2 to the absence of TAAR5. Venn diagrams and sample-specific breakdowns of composition by phyla are shown in Figure 2b,c. The community structure (Figure 2c) was dominated by the phyla *Bacteroidota* and *Bacillota* in both groups. At the genus level (Figure 3a), however, KO mice exhibited distinct compositional shifts. Differential abundance analysis identified several affected taxa in KO mice (FDR < 0.05) (Supplementary Table S1). Notably, KO mice showed significant reductions in *Ruminococcus* and ASF356 (Figure 3b).

### 2.3. Predicted Metabolic Changes in TAAR5-KO Gut Microflora

Functional prediction using PICRUSt2 and the KEGG pathway database indicated that the taxonomic shifts in the mutants were associated with significant metabolic changes given the inferred metagenome. The PICRUSt2 prediction pipeline cross-references gene copy numbers in the inferred metagenome with the KEGG pathway database of gene sets annotated as parts of various metabolic pathways. As such, its ‘results’ are data-driven hypotheses (i.e., mere predictions based on plausible copy numbers); they are not conclusions. The PICRUSt2 pipeline predicted a marked decrease in the metagenomic capacity for central metabolic pathways in the gut microbiota of TAAR5-KO mice (Figure 4a). These included glycolysis, the tricarboxylic acid (TCA) cycle, fatty acid β-oxidation, and nucleotide biosynthesis, all of which contribute to the supply of accessible energy in microbes. In contrast, the metagenomic capacities of PWY-6595 and PWY-6607 were the only two pathways predicted to significantly increase in TAAR5-KO samples. Both annotations refer to a pathway wherein guanosine monophosphate is degraded stepwise to produce ammonium and urate. To assess the quality of these predictions, we followed the recommended guidelines on the interpretation of the weighted Nearest Sequenced Taxon Index (NSTI), which is the median distance from the sequenced amplicon to its closest counterpart in the reference database. The NSTI values did not significantly differ across genotypes (*p* = 0.62), with median values of 0.148 for WT and 0.143 for KO, both below the recommended threshold of 0.15 (Figure 4b).

### 2.4. Differential Abundance Analysis Reveals Significant Taxonomic Shifts in TAAR5-KO Mice

To visualize the global pattern of taxon-level shifts, we performed differential abundance analysis using ANCOM-BC2 with FDR correction. The volcano plot (Figure 5) summarizes the distribution of log2 fold changes and statistical significance for all genera. This analysis confirmed that the most pronounced changes in TAAR5-KO mice were the significant reduction in *Ruminococcus* (logFC = −4.934, FDR = 2.85 × 10^−6^), Rikenella (logFC = −2.914, FDR = 0.029), and ASF356 (logFC = −2.717, FDR = 0.029). Conversely, several taxa were enriched in KO mice, including Lachnospiraceae_NK4A136_group (logFC = +3.870, FDR = 0.009) and Prevotellaceae_UCG-001 (logFC = +3.921, FDR = 0.029). The overall distribution of points shows a skew towards taxa enriched in WT mice (blue), indicating that TAAR5 deletion primarily leads to the loss of specific microbial groups rather than the gain of new ones.

### 2.5. In Silico Docking Identifies Candidate Microbial Metabolites as Putative TAAR5 Ligands

To explore potential functional links between the altered microbiota and TAAR5, we performed molecular docking of 666 metabolites corresponding to significantly altered ECs (PICRUSt2, FDR < 0.05). Given the known size bias of AutoDock Vina, we used ligand efficiency (LE = −Vina/HAC) as the primary ranking metric (Supplementary Figure S1). Using LE for ranking, we identified three small amine compounds with LE values comparable to trimethylamine (TMA, LE = 0.596), the known endogenous TAAR5 agonist (Figure 6a, Table 1). The top candidates included 2-aminophenol (LE = 0.635), aminoacetone (LE = 0.664), and butan-1-amine (LE = 0.731). Notably, the ECs corresponding to 2-aminophenol and aminoacetone showed opposite trends: 2-aminophenol was decreased in KO (logFC = −4.77, FDR < 0.05), while aminoacetone was increased (logFC = +1.03, FDR < 0.05), and butan-1-amine corresponded to an EC that was downregulated (logFC = −4.75, FDR < 0.05). To validate our docking protocol, we analyzed the predicted binding poses of known TAAR5 ligands (TMA, DMEA, α-NETA). For all three compounds, the amine group formed a stable interaction with the conserved Asp114 residue in transmembrane helix 3 (TM3), and additional hydrophobic contacts with Phe196 and Trp200 (TM5) were consistently observed (Supplementary Figure S3).

In addition, all seven known TAAR5 ligands used as positive controls ranked above negative controls in terms of LE, with TMA showing the highest LE (0.596) despite its small size (Supplementary Tables S3 and S4). The predicted binding poses of the three candidate compounds (Figure 6b; Supplementary Figure S2, Supplementary Table S2) showed that all occupy the orthosteric pocket of mTAAR5. The amine group of each ligand interacts with Asp114, forming a salt bridge or hydrogen bond, while the hydrophobic moieties engage with Phe196 and Trp200.

Shown are compounds with ligand efficiency (LE = −Vina/HAC) ≥ 0.6, comparable to trimethylamine (TMA, LE = 0.596), the known endogenous TAAR5 agonist. EC numbers correspond to enzymes significantly altered in TAAR5-KO microbiota (FDR < 0.05). logFC values indicate KO minus WT; positive values correspond to ECs enriched in KO mice. HAC: heavy atom count; LE: ligand efficiency.

## 3. Discussion

Deletion of the murine TAAR5 increases the variability of gut microbial communities across individuals. Indeed, four of the eight mutants formed a separate cluster, and despite two being indistinguishable from WT, yet another two mutants had entirely unique compositions. This suggests that TAAR5 participates in the immune machinery which monitors and controls gut microbial composition. The absence of TAAR5 seems to decrease the abundances of *Turicimonas*, *Ruminococcus*, *Rikenella*, and ASF356. The most pronounced and statistically significant change was the reduction in *Ruminococcus* (logFC = −4.934, FDR = 2.85 × 10^−6^). Reductions were also observed for *Rikenella* and ASF356, but to a lesser extent. This is the first evidence linking TAAR5 to *Ruminococcus* abundance (Figure 3b).

Several *Ruminococcus* spp. produce TMA from dietary choline, so the observed result may reflect a feedback loop between TAAR5 activity and the abundances of *Ruminococcus* spp. As of 15/05/2026, looking up the TMA-producing bacterial enzyme CutC on UniProtKB yields 37 hits for *Ruminococcus*, two hits for *Rikenella*, and no hits for either *Turicimonas* or ASF356. Thus, the idea that TMA biosynthesis undergoes some feedback from TAAR5 may parsimoniously explain why *Ruminococcus* stands out among the significantly affected genera.

Crucially, a pioneering study has recently demonstrated that TAAR5 exerts a broad range of circadian effects in mice, including shifts in gut microbial composition [37]. Among the many variables assayed was the concentration of TMA in plasma (see Supplement Figure S1 therein). The trend shows that in the late dark phase, TMA concentrations dip in WT but not TAAR5-KO mice. This did not reach significance, likely due to sample sizes of *n* = 3–6.

Since many *Ruminococcus* spp. produce TMA, and since the abundance of *Ruminococcus* in TAAR5-KOs is reduced, one would think that plasma TMA levels would decrease in the mutants. Here, it is important to return to the periodic transcription of TAAR5; we had independently postulated this for the TAARs in our prior publications [38,39], and since found that others had already confirmed it in muscle tissue. Indeed, in a 2022 study in mice, Schugar and colleagues established that TAAR5 transcripts in cardiac and skeletal muscle are predominantly expressed in the circadian dark phase, with peak expression detected at the 22nd hour of Zeitgeber time (ZT22) [40]. As it happens, the non-significant dip in plasmatic TMA coincides with this at ZT22 [37], and in both papers, the samples were taken at four-hour intervals. It is possible that TAAR5 expression similarly oscillates in the immune cells that patrol the gut. Indeed, circadian oscillations of many genes tend to synchronize across tissues; several circadian effects occur in the immune system, including gene expression, cell maturation, migration, and antibody secretion.

In line with this explanation, our results show that the variance in the abundances of *Ruminococcus* was about twice as large in WT as it was in TAAR5-KO. This would make sense under the model we propose, as the abundance of *Ruminococcus* species would oscillate as a function of TAAR5′s circadian expression in WT but not in mutant mice. Our study was not designed with the possibility of circadian effects in mind, and as such, the fecal samples would have captured the gut microbiota at various time points in the cycle. However, the putative circadian oscillation of TAAR5 transcription in the immune cells of the gut does not account for the reduction in *Ruminococcus* in mutants. This reduction may have arisen due to second-order effects such as microbial competition for ecological niches or the like. Indeed, the range of possible microbial compositions is much wider in the absence of TAAR5, and the average reduction in *Ruminococcus* may simply be due to this, bearing only an indirect link to the loss of a TAAR5-dependent immune effect.

The consequent change in the microbial metabolome may also exert immune effects of their own. In a post hoc attempt to infer the physiological significance of the gut microbial shifts as a whole, we generated metagenomic predictions using the PICRUSt2 pipeline. Most of the predictions suggested altered energy utilization in the microbes, but the most interesting prediction was the increased capacity for ammonium and urate synthesis. These predictions should be interpreted with caution, as PICRUSt2 infers metabolic potential from 16S rRNA data and does not measure pathway activity or metabolite production. Nevertheless, these predictions would implicate the immune system if true, as both ammonium and urate ultimately stimulate NLRP3 inflammasomes downstream [41,42,43]. If these predictions are true, the mechanisms would relate to the known effects of *Ruminococcus* species in gut inflammation [44,45], which would bear clinical relevance to irritable bowel disease (i.e., both Crohn’s disease and ulcerative colitis) [46]. However, considering the wide variance of the reductions in *Ruminococcus*, and the lack of circadian tracking, a conclusion on the directionality of TAAR5′s effects on *Ruminococcus* may be premature, especially in humans.

The identification of three small amine compounds—2-aminophenol, aminoacetone, and butan-1-amine—as candidate TAAR5 ligands is particularly intriguing because their corresponding ECs were significantly altered in TAAR5-KO microbiota. This suggests a potential feedback loop: the absence of TAAR5 may relieve negative regulation of bacterial pathways that produce their own ligands. The fact that 2-aminophenol (predicted to be decreased) and aminoacetone (predicted to be increased) in KO showed ligand efficiencies exceeding those of TMA further supports their plausibility as candidates.

However, we emphasize that these findings are strictly computational and should thus be understood as hypotheses rather than conclusions. The validation on known TAAR5 ligands confirmed that our docking protocol captures the essential biophysics of ligand–receptor recognition, as evidenced by the recapitulation of canonical interactions with Asp114, Phe196, and Trp200. Nevertheless, future studies are required to experimentally validate the binding and functional activity of the candidate compounds using in vitro assays (e.g., BRET-based cAMP measurements) and to confirm their in vivo presence and concentration changes by targeted metabolomics.

## 4. Materials and Methods

### 4.1. Animals and Sample Collection

This study was carried out in compliance with the guidelines of the Ministry of Health in the Russian Federation, and the principles adopted by the FELASA and RusLASA organizations on the welfare of laboratory animals. The protocol was approved by the Saint Petersburg State University Ethical Committee for Animal Research (131-03-1 of 8 January 2024). Male mice (12 weeks old) were housed in groups of three or four per cage and maintained under standard conditions (12 h light/dark cycle, 21 ± 1 °C, and 40–70% humidity) with food and water provided ad libitum. Fecal samples were collected from TAAR5-KO mice (*n* = 8) and WT littermates (*n* = 12).

### 4.2. Gut Bacterial DNA Extraction and Sequencing

Fecal samples (100 mg) were used for DNA extraction with the Hipure Soil DNA Kit (Magen Biotech, Guangzhou, China) according to the manufacturer’s protocol. The 16S ribosomal RNA (rRNA) gene was amplified with the primers (positions based on E. coli SSU rRNA numbering): 16S F 343-CCTACGGGNGGCWGCAG and 16S R 806-GGACTACHVGGGTATCTAATCC. The primers target the V3-V4 hypervariable region of bacterial 16S rRNA genes. Polymerase chain reactions (PCRs) were then performed under the following conditions: denaturation (95 °C, 3 min), 25 cycles of denaturation (95 °C, 30 s), annealing (55 °C, 30 s), elongation (72 °C, 30 s), and a final elongation (72 °C, 5 min). Sequencing was performed on the Illumina HiSeq 2000 platform (San Diego, CA, USA).

### 4.3. Microbial 16S rRNA Gene Analysis

FASTQ-formatted raw sequence data were processed in the QIIME2 environment (v2023.5) using the DADA2 v1.32.0 plugin for read error correction, quality filtering at Q > 25, denoising, merging, chimera removal, and amplicon sequence variant (ASV) table generation [47]. Taxonomic classification was performed using a Naïve Bayes classifier trained on all available variants of the V3–V4 region in the SILVA 138.1 high-quality rRNA database [48]. For phylogenetic diversity analysis, an aligned tree was constructed using MAFFT v7.505 and FastTree v2.1.11. All outputs (ASV table, taxonomy, tree, metadata) were imported into R 4.5.0 using the qiime2R package (v0.2.0) for subsequent analysis. Bioinformatic processing in R involved creating a phyloseq object and calculating diversity metrics including traditional indices (Shannon, Simpson, Pielou) and Hill numbers (q = 0, 1, 2), as well as Bray–Curtis dissimilarities using the phyloseq (v1.52.0) and MicrobiotaProcess packages (v1.20.2).

### 4.4. Functional Metagenome Prediction

Phylogenetic investigation of communities by reconstruction of unobserved states (PICRUSt2) (v2.6.3) [49] was used to predict the functional potential of the bacterial communities from the ASV table. The predicted metagenome was annotated against databases of the Enzyme Commission (EC) [50] numbers and KEGG pathways [51]. We used these annotations to identify differentially abundant enzyme genes in the KO vs. WT metagenomes to specify the metabolic pathways affected by TAAR5 deletion. PICRUSt2 predictions represent inferred metagenomic content based on 16S rRNA gene copy numbers and reference genomes; they do not measure transcriptional activity or metabolic flux. Accordingly, all functional predictions are presented as inferred capacities for KEGG pathways rather than demonstrated activity. In this sense, PICRUSt2 is just an automated method of generating data-driven hypotheses.

### 4.5. Microbiome Community and Statistical Analysis

Statistical analyses and plotting were performed in R using the phyloseq (1.52.0) [52], MicrobiotaProcess (1.20.2) [53], vegan (2.7-2) [54], pheatmap (1.0.13) [55], edgeR (4.6.3) [56], and limma (3.64.3) [57] packages. Prior to all downstream analyses, ASVs with a relative abundance below 0.25% across all samples were removed to reduce noise from spurious taxa. Alpha-diversity metrics, including traditional indices (Shannon, Simpson, Pielou) and Hill numbers (q = 0, 1, 2), were calculated in R (4.5). Hill numbers represent effective numbers of species: q = 0 corresponds to observed richness (ASV count), q = 1 to exponential Shannon, and q = 2 to inverse Simpson. Beta-diversity was assessed using Bray–Curtis dissimilarities. Principal Coordinate Analysis (PCoA) based on Bray–Curtis dissimilarity was used to visualize sample similarity. Within- and between-group dissimilarity comparisons were performed using the same metric. To account for potential cage effects, PERMANOVA was also performed with cage as a stratification factor; the genotype effect remained significant (*p* < 0.01). For taxonomic differential abundance at the genus level, we used ANCOM-BC2 (v2.0.2), which estimated the bias-corrected log fold change between KO and WT mice, with positive coefficients indicating higher abundance in KO mice and negative coefficients indicating lower abundance in KO mice. Raw *p*-values were adjusted for multiple testing using the Benjamini–Hochberg procedure. Genera with an adjusted q-value < 0.05 were considered statistically significant. Genera with 0.05 ≤ q < 0.10 were additionally displayed as suggestive associations in visualizations. For visualization, genera with q < 0.10 were presented using paired panels showing mean relative abundance in WT and KO samples and the corresponding ANCOM-BC2 log fold-change estimate with its 95% confidence interval. For functional metagenomic predictions (PICRUSt2), differential abundance of KEGG pathways was assessed using limma-voom with Benjamini–Hochberg correction, as implemented in the limma package. Taxa were labelled at the family–genus level where possible to facilitate interpretation of incompletely resolved genera. Due to the limited sample size (*n* = 8), KO subgroups were evaluated heuristically without parametric inference.

### 4.6. In Silico Docking of Microbial Metabolites Against Murine TAAR5

In silico docking was performed to explore potential ligand-receptor interactions between microbial metabolites and murine TAAR5. A total of 666 small molecules corresponding to significantly altered EC numbers identified by PICRUSt2 (FDR < 0.05) were docked. Ligands were retrieved from the BRENDA database (v2024.1) and prepared using OpenBabel (v3.1.1) with MMFF94 minimization and protonation at physiological pH (7.4). A homology model of murine TAAR5 was generated using the Cryo-EM structure of human TAAR1 (PDB ID: 7XP9) as a template (SWISS-MODEL v2024.1, refined with Modeller v10.5). The binding pocket was defined around the conserved Asp114 residue with a 25 × 25 × 25 Å search box. Docking was performed with AutoDock Vina (v1.2.0) using exhaustiveness = 32 and 30 independent poses per ligand. Known TAAR5 ligands (TMA, α-NETA, 8-OH-DPAT, BRIL-54443, RS 23597, SDZ 205-557) were included as positive controls, and a set of 11 inactive compounds served as negative controls (Supplementary Table S4). Ligand efficiency (LE = −Vina_score/Heavy Atom Count) was applied as the primary ranking metric to account for the size bias of AutoDock Vina (Supplementary Figure S1). For docking protocol validation, the predicted binding poses of known TAAR5 ligands (TMA, DMEA, α-NETA) were analyzed. The amine group was required to form a stable interaction with Asp114 (TM3), with additional hydrophobic contacts involving Phe196 and Trp200 (TM5). All positive controls ranked above negative controls in terms of LE, confirming that the protocol captures the essential biophysics of ligand–receptor recognition (Supplementary Figure S3, Supplementary Tables S3 and S4). Binding poses were visualized with PyMOL (v2.5; Schrödinger, LLC, New York, NY, USA). The full docking protocol and extended validation are described in the Supplementary Methods.

## 5. Conclusions

The present study adds to the growing evidence that TAAR5 plays a role in shaping the composition of the mouse gut microbiota, and suggests that knockout of Taar5 expands the variability of microbial composition, with a striking reduction in Ruminococcus species. These results are most likely due to the function of TAAR5 in the immune cells of the gut, which regulate the gut microbiota via B-cell secretions trafficked into the gut lumen. *Ruminococcus* species in particular, and gut microbes in general, are critical actors in the pathophysiology of irritable bowel diseases. In this light, our study fits into a burgeoning literature that hints at TAAR5′s potential as an immunologically useful target for future treatments of gut disease and the psychiatric disorders they are so commonly comorbid with.

In addition, in silico docking identified three candidate microbial metabolites, 2-aminophenol, aminoacetone, and butan-1-amine, as putative TAAR5 ligands with ligand efficiencies comparable to TMA. These candidates correspond to ECs significantly altered in TAAR5-KO microbiota and provide testable hypotheses for future experimental validation.

## Figures and Tables

**Figure 1:**
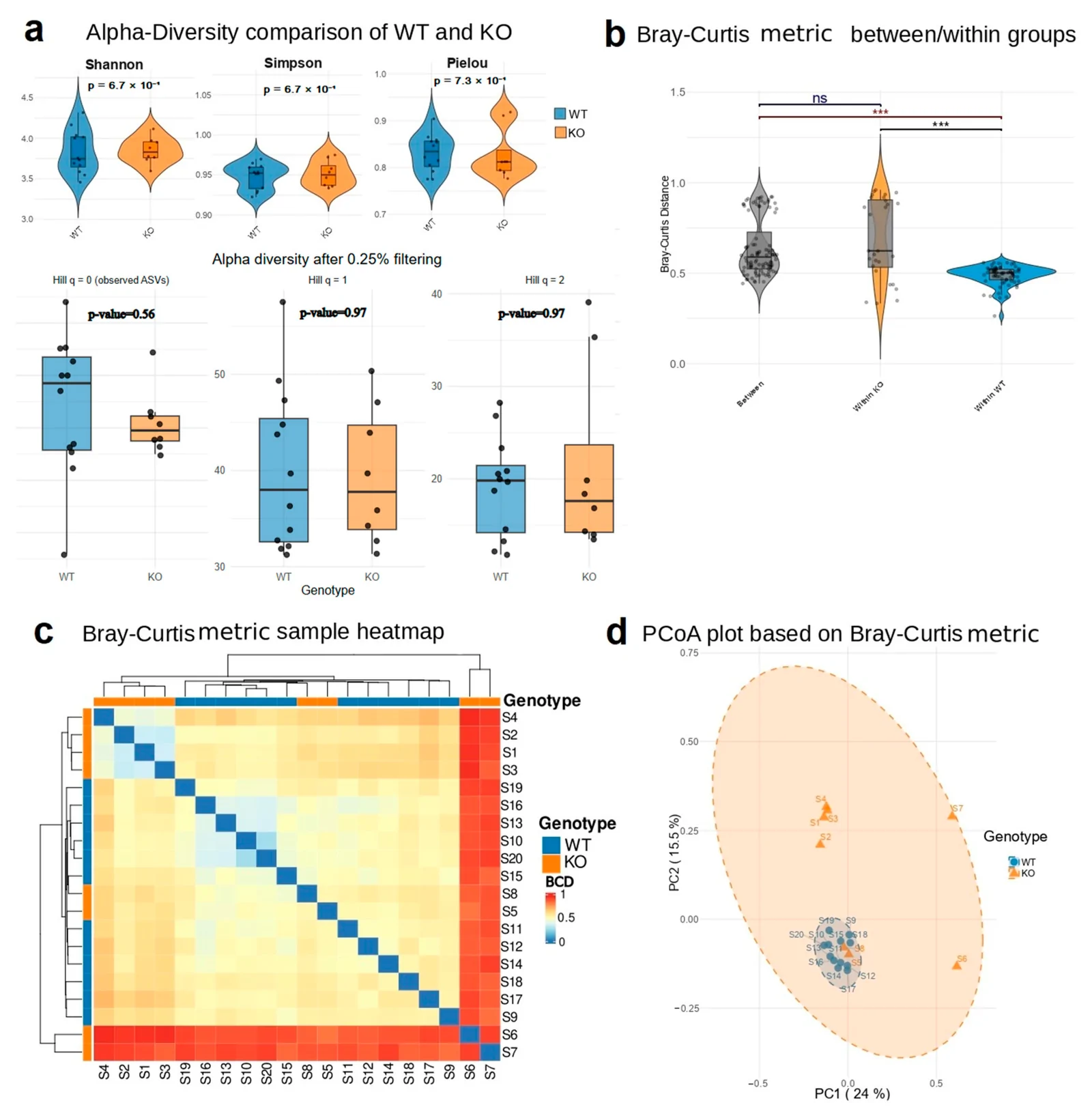
Alpha and Beta diversity of the gut microbiota in the TAAR5-KO and WT mice. (**a**) Alpha-diversity assessed using traditional indices (Shannon, Simpson, Pielou) and Hill numbers (q = 0, 1, 2), which represent effective numbers of species (q = 0—observed richness, q = 1—exponential Shannon, q = 2—inverse Simpson). No significant differences were observed between WT and KO groups (Mann–Whitney U test, *p* > 0.05 for all). (**b**) Violin plots depicting Bray–Curtis dissimilarities between samples within and outside of groups, Mann–Whitney U test; *** *p* < 0.001; ns, not significant. (**c**) Heatmap and hierarchical clustering between samples and hierarchical clustering trees of samples at the ASV level based on the Bray–Curtis method. Values were normalized to a range from 0 to 1. (**d**) Principal coordinate analysis (PCoA) of the gut microbiota using the Bray–Curtis β-diversity metric. Each point represents the composition of the fecal microbiota of one sample.

**Figure 2:**
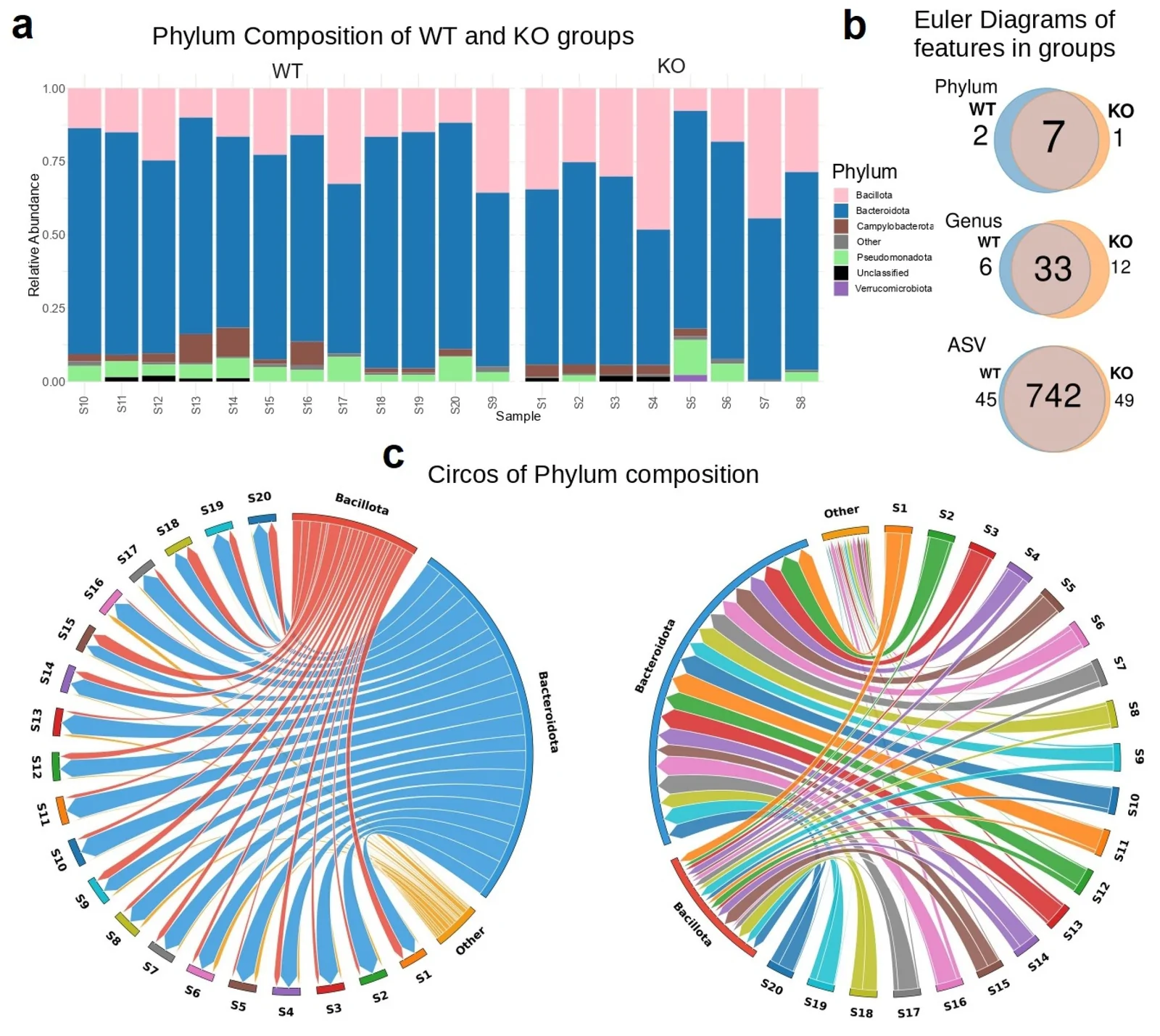
Taxonomic composition and shifts between TAAR5-KO and WT mice. (**a**) Bar plot of bacterial phyla; phyla with <1% relative abundance included under the “Other” label. (**b**) Venn diagrams depicting commonality of features in two experimental groups: two taxonomic features and ASVs (amplicon variants). (**c**) Chord diagrams depicting phylum distribution between samples—phyla other than Bacteroidota and Bacillota included under the “Other” label.

**Figure 3:**
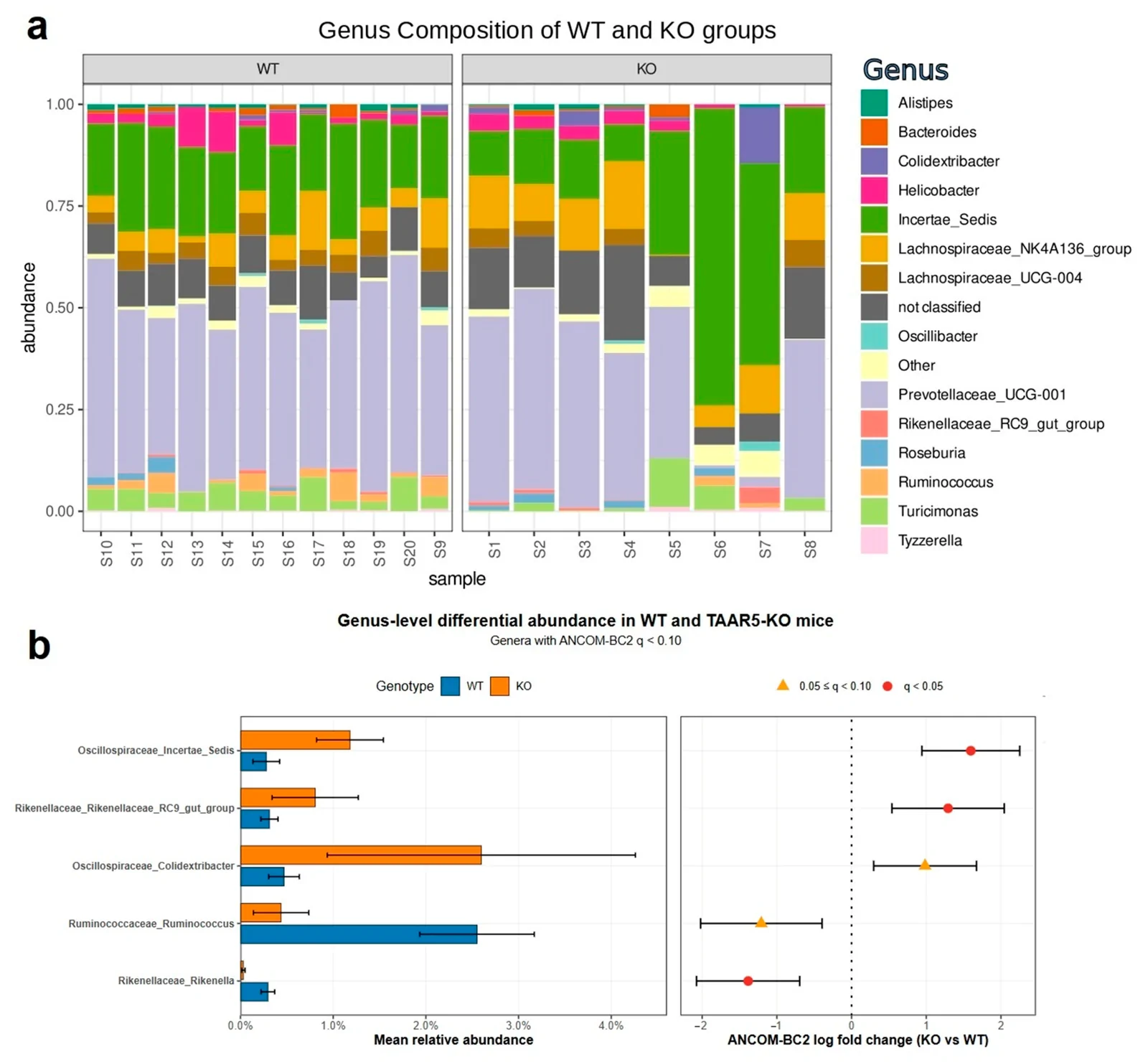
Taxonomic composition of gut microbiota between TAAR5-KO and WT mice. (**a**) Bar plot of bacterial genera; genera with <1% relative abundance included under the “Other” label. (**b**) Genera with significantly shifted abundance across the whole KO group vs. WT and 95% confidence intervals.The dotted vertical line in panel (**b**) indicates zero log fold change (no difference between KO and WT mice).

**Figure 4:**
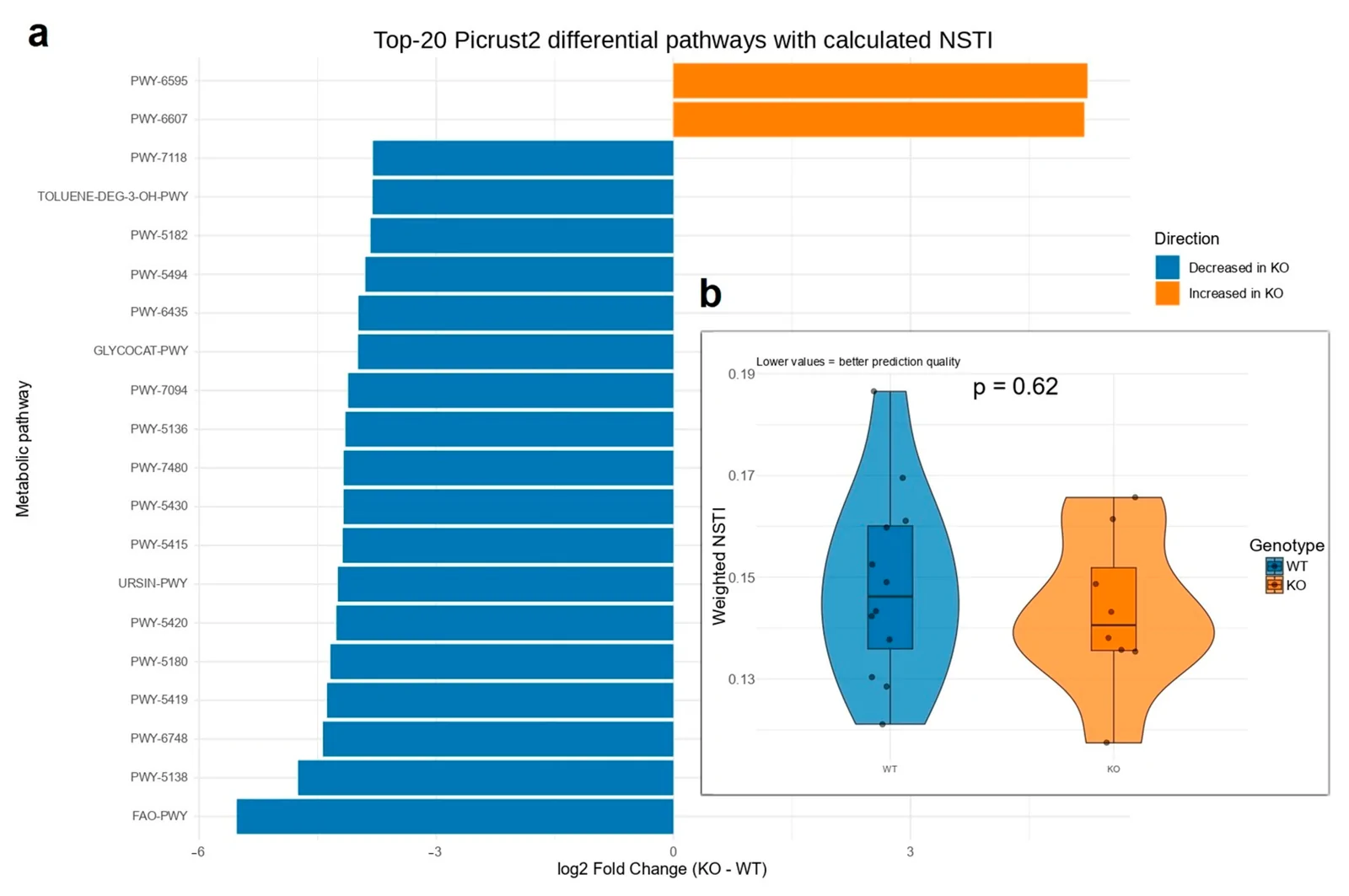
Predicted changes in microbial metabolic pathways in TAAR5-KO vs. WT mice. (**a**) Bar chart showing the log2 fold-change (KO/WT) for the top 20 significantly altered KEGG pathways (PICRUSt2, limma-voom, *p* < 0.05). Pathways are ranked by absolute fold-change. KO microbiota are predicted to have decreased capacity for core metabolic pathways (negative logFC) and increased capacity for specific degradation pathways (positive logFC): PWY-6607, PWY-6595. (**b**) NSTI (nearest sequenced taxon metric) of samples in experimental groups calculated by the PICRUSt2 pipeline. A lower metric indicates higher-quality prediction.

**Figure 5:**
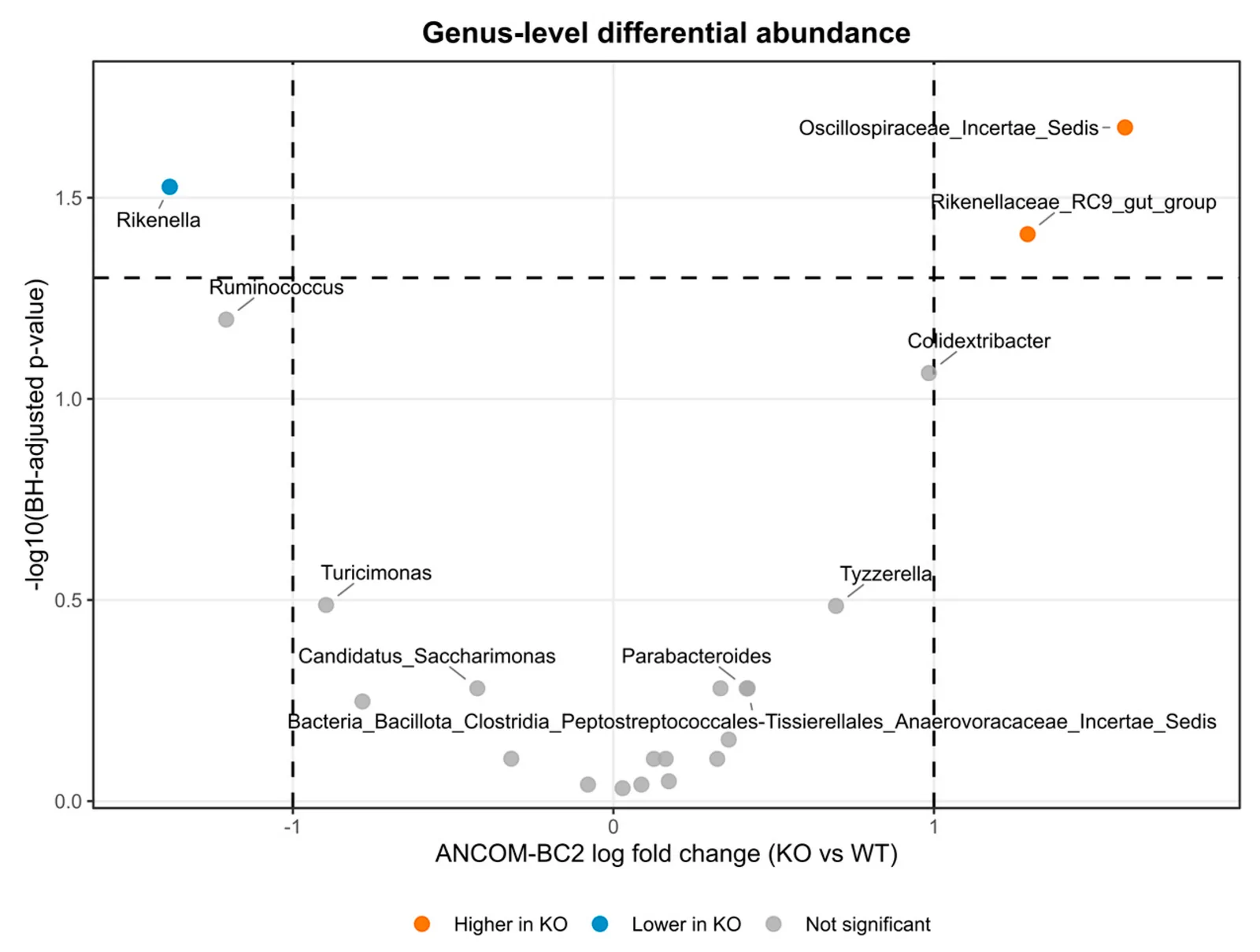
Volcano plot of differential abundance analysis of gut microbial genera between TAAR5-KO and WT mice. The *x*-axis represents the log2 fold change (logFC), calculated as KO minus WT; positive values indicate taxa enriched in KO mice, negative values indicate taxa enriched in WT mice. The *y*-axis shows the negative logarithm of the false discovery rate (-log10 FDR), highlighting taxa with the most statistically significant differences. Significantly altered genera (FDR < 0.05) are shown in colour: red for KO-enriched, blue for WT-enriched. The most prominent changes include reduction in *Ruminococcus*, *Rikenella*, and ASF356 in KO mice, and enrichment of specific *Lachnospiraceae* and *Prevotellaceae* lineages. Dashed lines indicate thresholds for significance (FDR = 0.05, horizontal) and fold change (|logFC| > 1, vertical).

**Figure 6:**
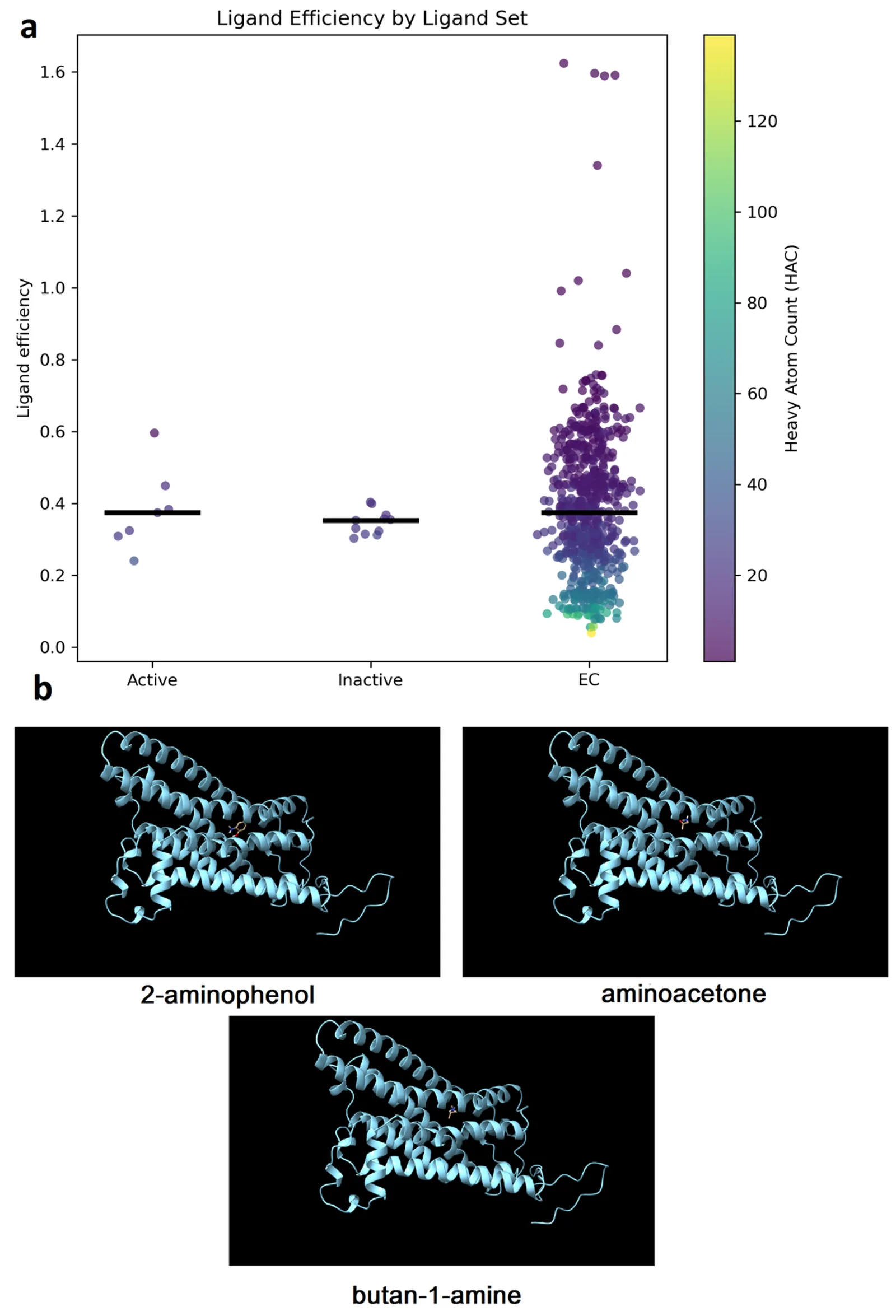
Identification of candidate microbial metabolites as putative TAAR5 ligands. (**a**) Ligand efficiency (LE = −Vina/HAC) of known TAAR5 ligands (grey) and candidate metabolites (blue). Known ligands: TMA (LE = 0.596), α-NETA (0.374), 8-OH-DPAT (0.383), BRIL-54443 (0.449), RS 23597 (0.309), SDZ 205-557 (0.324). Candidates: 2-aminophenol (0.635), aminoacetone (0.664), butan-1-amine (0.731). The dashed line indicates TMA LE (0.596). Error bars represent the standard deviation (*n* = 3 docking runs). (**b**) Predicted binding poses of the three candidate ligands within the murine TAAR5 homology model. From left to right: 2-aminophenol (BRENDA ID: 1910), aminoacetone (BRENDA ID: 8045), and butan-1-amine (BRENDA ID: 1381). Ligands are shown as sticks (carbon: green, oxygen: red, nitrogen: blue). Key interacting residues (Asp114, Phe196, Trp200) are shown as lines. The amine group of each ligand interacts with Asp114 (TM3); additional hydrophobic contacts involve Phe196 and Trp200 (TM5). Docking was performed using AutoDock Vina against a homology model of murine TAAR5 (based on the human TAAR1 Cryo-EM structure, PDB: 7XP9).

**Table 1:** Top candidate microbial metabolites identified by molecular docking.

Compound	EC Number(s)	logFC (KO/WT)	FDR	LE
2-aminophenol	4.1.1.77, 4.2.1.80	−4.768	0.048	0.635
Aminoacetone	4.1.2.48	+1.026	0.047	0.664
butan-1-amine	1.4.9.1	−4.747	0.048	0.731
TMA (reference)	-	-	-	0.596

## Data Availability

The original contributions presented in this study are included in the article/Supplementary Material. Further inquiries can be directed to the corresponding authors.

## Supplementary Materials

The following supporting information can be downloaded at: https://www.mdpi.com/article/10.3390/ijms27198493/s1.

## References

[1] Gainetdinov R.R., Hoener M.C., Berry M.D. (2018). Trace Amines and Their Receptors. Pharmacol. Rev..

[2] Guo L., Dai W., Xu Z., Liang Q., Miller E.T., Li S., Gao X., Baldwin M.W., Chai R., Li Q. (2022). Evolution of Brain-Expressed Biogenic Amine Receptors into Olfactory Trace Amine-Associated Receptors. Mol. Biol. Evol..

[3] Saraiva L.R., Riveros-McKay F., Mezzavilla M., Abou-Moussa E.H., Arayata C.J., Makhlouf M., Trimmer C., Ibarra-Soria X., Khan M., Van Gerven L. (2019). A Transcriptomic Atlas of Mammalian Olfactory Mucosae Reveals an Evolutionary Influence on Food Odor Detection in Humans. Sci. Adv..

[4] Qian K., Hao Y., Wang Z., Li Q. (2025). Ligand Recognition and Function of TAARs. Sens. Neurosci..

[5] Galstyan D.S., Krotova N.A., Lebedev A.S., Kotova M.M., Martynov D.D., Golushko N.I., Perederiy A.S., Zhukov I.S., Rosemberg D.B., Lim L.W. (2025). Trace Amine Signaling in Zebrafish Models: CNS Pharmacology, Behavioral Regulation and Translational Relevance. Eur. J. Pharmacol..

[6] Guo L., Cheng J., Lian S., Liu Q., Lu Y., Zheng Y., Zhu K., Zhang M., Kong Y., Zhang C. (2023). Structural Basis of Amine Odorant Perception by a Mammal Olfactory Receptor. Nature.

[7] Liberles S.D. (2015). Trace Amine-Associated Receptors: Ligands, Neural Circuits, and Behaviors. Curr. Opin. Neurobiol..

[8] Liberles S.D., Buck L.B. (2006). A Second Class of Chemosensory Receptors in the Olfactory Epithelium. Nature.

[9] Efimova E.V., Shemiakova T.S., Maslennikova D.D., Vaganova A.N., Markina A.A., Gainetdinov R.R. (2025). Effect of Chronic Unpredictable Mild Stress on TAAR2 Knockout Mice. Cell Tissue Biol..

[10] Efimova E.V., Kuvarzin S.R., Mor M.S., Katolikova N.V., Shemiakova T.S., Razenkova V., Ptukha M., Kozlova A.A., Murtazina R.Z., Smirnova D. (2022). Trace Amine-Associated Receptor 2 Is Expressed in the Limbic Brain Areas and Is Involved in Dopamine Regulation and Adult Neurogenesis. Front. Behav. Neurosci..

[11] Efimova E.V., Kozlova A.A., Razenkova V., Katolikova N.V., Antonova K.A., Sotnikova T.D., Merkulyeva N.S., Veshchitskii A.S., Kalinina D.S., Korzhevskii D.E. (2021). Increased Dopamine Transmission and Adult Neurogenesis in Trace Amine-Associated Receptor 5 (TAAR5) Knockout Mice. Neuropharmacology.

[12] Shemiakova T.S., Markina A.A., Efimova E.V., Murtazina R.Z., Volnova A.B., Veshchitskii A.A., Leonova E.I., Gainetdinov R.R. (2025). TAAR8 in the Brain: Implications for Dopaminergic Function, Neurogenesis, and Behavior. Biomedicines.

[13] Park S.J., Greer P.L., Lee N. (2024). From Odor to Oncology: Non-Canonical Odorant Receptors in Cancer. Oncogene.

[14] Pitts M.S., McShane J.N., Hoener M.C., Christian S.L., Berry M.D. (2019). TAAR1 Levels and Sub-Cellular Distribution Are Cell Line but Not Breast Cancer Subtype Specific. Histochem. Cell Biol..

[15] Vaganova A.N., Maslennikova D.D., Konstantinova V.V., Kanov E.V., Gainetdinov R.R. (2023). The Expression of Trace Amine-Associated Receptors (TAARs) in Breast Cancer Is Coincident with the Expression of Neuroactive Ligand–Receptor Systems and Depends on Tumor Intrinsic Subtype. Biomolecules.

[16] Panas M.W., Xie Z., Panas H.N., Hoener M.C., Vallender E.J., Miller G.M. (2012). Trace Amine Associated Receptor 1 Signaling in Activated Lymphocytes. J. Neuroimmune Pharmacol..

[17] Babusyte A., Kotthoff M., Fiedler J., Krautwurst D. (2013). Biogenic Amines Activate Blood Leukocytes via Trace Amine-Associated Receptors TAAR1 and TAAR2. J. Leukoc. Biol..

[18] Murtazina R.Z., Zhukov I.S., Korenkova O.M., Popova E.A., Kuvarzin S.R., Efimova E.V., Kubarskaya L.G., Batotsyrenova E.G., Zolotoverkhaya E.A., Vaganova A.N. (2021). Genetic Deletion of Trace-Amine Associated Receptor 9 (TAAR9) in Rats Leads to Decreased Blood Cholesterol Levels. Int. J. Mol. Sci..

[19] Kim J.B., Bae J.E., Park N.Y., Kim Y.H., Kim S.H., Hyung H., Yeom E., Choi D.K., Jeong K., Cho D.H. (2024). TAAR8 Mediates Increased Migrasome Formation by Cadaverine in RPE Cells. Curr. Issues Mol. Biol..

[20] Zhukov I.S., Kubarskaya L.G., Karpova I.V., Vaganova A.N., Karpenko M.N., Gainetdinov R.R. (2021). Minor Changes in Erythrocyte Osmotic Fragility in Trace Amine-Associated Receptor 5 (Taar5) Knockout Mice. Int. J. Mol. Sci..

[21] Berry M.D., Gainetdinov R.R., Hoener M.C., Shahid M. (2017). Pharmacology of Human Trace Amine-Associated Receptors: Therapeutic Opportunities and Challenges. Pharmacol. Ther..

[22] O’Donnell M.P., Fox B.W., Chao P.H., Schroeder F.C., Sengupta P. (2020). A Neurotransmitter Produced by Gut Bacteria Modulates Host Sensory Behaviour. Nature.

[23] Bugda Gwilt K., González D.P., Olliffe N., Oller H., Hoffing R., Puzan M., El Aidy S., Miller G.M. (2020). Actions of Trace Amines in the Brain-Gut-Microbiome Axis via Trace Amine-Associated Receptor-1 (TAAR1). Cell Mol. Neurobiol..

[24] Wallrabenstein I., Kuklan J., Weber L., Zborala S., Werner M., Altmüller J., Becker C., Schmidt A., Hatt H., Hummel T. (2013). Human Trace Amine-Associated Receptor TAAR5 Can Be Activated by Trimethylamine. PLoS ONE.

[25] Ito J., Ito M., Nambu H., Fujikawa T., Tanaka K., Iwaasa H., Tokita S. (2009). Anatomical and Histological Profiling of Orphan G-Protein-Coupled Receptor Expression in Gastrointestinal Tract of C57BL/6J Mice. Cell Tissue Res..

[26] Kalinina D.S., Ptukha M.A., Goriainova A.V., Merkulyeva N.S., Kozlova A.A., Murtazina R.Z., Shemiakova T.S., Kuvarzin S.R., Vaganova A.N., Volnova A.B. (2021). Role of the Trace Amine Associated Receptor 5 (TAAR5) in the Sensorimotor Functions. Sci. Rep..

[27] Massey W., Mukherjee P., Mrdjen M., Banerjee S., Burrows A., Wang Z., Brown J.M., Rieder F. (2025). Microbial Trimethylamine Host Trace Amine Associated Receptor 5 (Taar5) Signaling Exerts Pro-Fibrotic Properties in Ibd. Inflamm. Bowel Dis..

[28] Rivera C.A., Lennon-Duménil A.M. (2023). Gut Immune Cells and Intestinal Niche Imprinting. Semin. Cell Dev. Biol..

[29] Mörbe U.M., Jørgensen P.B., Fenton T.M., von Burg N., Riis L.B., Spencer J., Agace W.W. (2021). Human Gut-Associated Lymphoid Tissues (GALT); Diversity, Structure, and Function. Mucosal Immunol..

[30] Sterlin D., Fadlallah J., Slack E., Gorochov G. (2020). The Antibody/Microbiota Interface in Health and Disease. Mucosal Immunol..

[31] Yoo J.S., Oh S.F. (2023). Unconventional Immune Cells in the Gut Mucosal Barrier: Regulation by Symbiotic Microbiota. Exp. Mol. Med..

[32] Pabst O., Nowosad C.R. (2023). B Cells and the Intestinal Microbiome in Time, Space and Place. Semin. Immunol..

[33] Natvig I.B., Johansen F.E., Nordeng T.W., Haraldsen G., Brandtzaeg P. (1997). Mechanism for Enhanced External Transfer of Dimeric IgA over Pentameric IgM: Studies of Diffusion, Binding to the Human Polymeric Ig Receptor, and Epithelial Transcytosis. J. Immunol..

[34] Zhukov I.S., Vaganova A.N., Murtazina R.Z., Alferova L.S., Ermolenko E.I., Gainetdinov R.R. (2022). Gut Microbiota Alterations in Trace Amine-Associated Receptor 9 (TAAR9) Knockout Rats. Biomolecules.

[35] Vaganova A.N., Zhukov I.S., Shemiakova T.S., Rozhkov K.A., Alferova L.S., Karaseva A.B., Ermolenko E.I., Gainetdinov R.R. (2024). Functional Analysis of TAAR1 Expression in the Intestine Wall and the Effect of Its Gene Knockout on the Gut Microbiota in Mice. Int. J. Mol. Sci..

[36] Nicoli A., Weber V., Bon C., Steuer A., Gustincich S., Gainetdinov R.R., Lang R., Espinoza S., Di Pizio A. (2023). Structure-Based Discovery of Mouse Trace Amine-Associated Receptor 5 Antagonists. J. Chem. Inf. Model..

[37] Mahen K.K., Massey W.J., Orabi D., Brown A.L., Jaramillo T.C., Burrows A., Horak A.J., Dutta S., Mrdjen M., Mouannes N. (2026). Gut Microbe-Derived Trimethylamine Shapes Circadian Rhythms through the Host Receptor TAAR5. eLife.

[38] Alnefeesi Y., Murtazina R.Z., Gainetdinov R.R. (2025). Trace Amine-Associated Receptors (TAARs): Candidate Targets in the Treatment of Bipolar Disorders. Actas Esp. Psiquiatr..

[39] Zhukov I.S., Karpova I.V., Murtazina R.Z., Alnefeesi Y., Korenkova O.M., Tissen I.Y., Palchikova S.A., Tokareva L.A., Pyurveev S.S., Shabanov P.D. (2025). TAAR9 Knockout Increases Hippocampal Serotonin and Alters Grooming Behavior in Rats. Front. Pharmacol..

[40] Schugar R.C., Gliniak C.M., Osborn L.J., Massey W., Sangwan N., Horak A., Banerjee R., Orabi D., Helsley R.N., Brown A.L. (2022). Gut Microbe-Targeted Choline Trimethylamine Lyase Inhibition Improves Obesity via Rewiring of Host Circadian Rhythms. eLife.

[41] Cheon S.Y., Kim M.Y., Kim J., Kim E.J., Kam E.H., Cho I., Koo B.N., Kim S.Y. (2023). Hyperammonemia Induces Microglial NLRP3 Inflammasome Activation via Mitochondrial Oxidative Stress in Hepatic Encephalopathy. Biomed. J..

[42] Cabău G., Crișan T.O., Klück V., Popp R.A., Joosten L.A.B. (2020). Urate-Induced Immune Programming: Consequences for Gouty Arthritis and Hyperuricemia. Immunol. Rev..

[43] Li D., Yuan S., Deng Y., Wang X., Wu S., Chen X., Li Y., Ouyang J., Lin D., Quan H. (2023). The Dysregulation of Immune Cells Induced by Uric Acid: Mechanisms of Inflammation Associated with Hyperuricemia and Its Complications. Front. Immunol..

[44] Henke M.T., Kenny D.J., Cassilly C.D., Vlamakis H., Xavier R.J., Clardy J. (2019). Ruminococcus Gnavus, a Member of the Human Gut Microbiome Associated with Crohn’s Disease, Produces an Inflammatory Polysaccharide. Proc. Natl. Acad. Sci. USA.

[45] Valentino V., De Filippis F., Marotta R., Pasolli E., Ercolini D. (2024). Genomic Features and Prevalence of Ruminococcus Species in Humans Are Associated with Age, Lifestyle, and Disease. Cell Rep..

[46] Silva I., da Silva M.T.B. (2025). Inflammatory Bowel Disease (IBD): Clinical Diagnosis and Treatment. J. Clin. Med..

[47] Callahan B.J., McMurdie P.J., Rosen M.J., Han A.W., Johnson A.J.A., Holmes S.P. (2016). DADA2: High-Resolution Sample Inference from Illumina Amplicon Data. Nat. Methods.

[48] Pruesse E., Peplies J., Glöckner F.O. (2012). SINA: Accurate High-Throughput Multiple Sequence Alignment of Ribosomal RNA Genes. Bioinformatics.

[49] Douglas G.M., Maffei V.J., Zaneveld J.R., Yurgel S.N., Brown J.R., Taylor C.M., Huttenhower C., Langille M.G.I. (2020). PICRUSt2 for Prediction of Metagenome Functions. Nat. Biotechnol..

[50] McDonald A.G., Boyce S., Tipton K.F. (2015). Enzyme Classification and Nomenclature. Encyclopedia of Life Sciences.

[51] Kanehisa M., Furumichi M., Sato Y., Matsuura Y., Ishiguro-Watanabe M. (2025). KEGG: Biological Systems Database as a Model of the Real World. Nucleic Acids Res..

[52] McMurdie P.J., Holmes S. (2013). Phyloseq: An R Package for Reproducible Interactive Analysis and Graphics of Microbiome Census Data. PLoS ONE.

[53] Xu S., Zhan L., Tang W., Wang Q., Dai Z., Zhou L., Feng T., Chen M., Wu T., Hu E. (2023). MicrobiotaProcess: A Comprehensive R Package for Deep Mining Microbiome. Innovation.

[54] Oksanen J., Simpson G.L., Blanchet F.G., Kindt R., Legendre P., Minchin P.R., O’Hara R.B., Solymos P., Stevens M.H.H., Szoecs E. (2001). Vegan: Community Ecology Package 2001.

[55] Kolde R. (2010). Pheatmap: Pretty Heatmaps, 2010.

[56] Chen Y., Chen L., Lun A.T.L., Baldoni P.L., Smyth G.K. (2025). EdgeR v4: Powerful Differential Analysis of Sequencing Data with Expanded Functionality and Improved Support for Small Counts and Larger Datasets. Nucleic Acids Res..

[57] Ritchie M.E., Phipson B., Wu D., Hu Y., Law C.W., Shi W., Smyth G.K. (2015). Limma Powers Differential Expression Analyses for RNA-Sequencing and Microarray Studies. Nucleic Acids Res..

