# Supplementary material for: Gut Microbiome Alterations in TAAR5 Knockout Mice and In Silico Ligand Prediction
Source: Int J Mol Sci. 2026 Sep 23;27(19):8493. doi: 10.3390/ijms27198493 (PMC13664747; doi:10.3390/ijms27198493)
Supplement: Supplementary file 1 [file ijms-27-08493-s001.zip › ijms-4513483-supplementary.pdf]

Supplementary Methods: Docking Score Validation and Ligand Efficiency Rationale

This section describes the validation of the docking protocol and the rationale for using ligand efficiency as the primary ranking metric. The construction of the murine TAAR5 homology model and the preparation of the ligand dataset are described in the main Materials and Methods.

Authors: Nikita S. Gladyshev, Alexey Zolkin

Ligand dataset and docking. A total of 666 unique small molecules corresponding to significantly altered ECs (PICRUSt2, FDR < 0.05) were retrieved from BRENDA (2024.1) and prepared for docking using OpenBabel (MMFF94 minimization, protonation at pH 7.4). Docking was performed with AutoDock Vina (v1.2.0) against the mTAAR5 homology model (based on human TAAR1 Cryo-EM structure, PDB: 7XP9). The search box was centered on the conserved aspartate (D114) with dimensions 25×25×25 Å; exhaustiveness was set to 32, and 30 independent poses were generated per ligand. Known TAAR5 ligands (TMA, α-NETA, 8-OH-DPAT, BRIL-54443, RS 23597, SDZ 205-557) were included as positive controls.

Size bias in AutoDock Vina scoring. During initial analysis, we observed a systematic bias in raw Vina scores: larger molecules (with higher heavy-atom count, HAC) consistently received more favourable (more negative) scores (**Supplementary Fig. S1a**). This size bias is a known limitation of AutoDock Vina and prompted us to assess the reliability of the scoring function.

Correlation with experimental activity. To evaluate whether raw Vina scores predict actual ligand activity, we compared them with experimental pEC50 values for known TAAR5 ligands. Theoretically, Vina score represents predicted binding energy, and the relationship with pEC50 can be expressed as:

$$pEC_{50} = -\log_{10}(EC_{50}) \quad \text{(S1)}$$

$$\Delta G^\circ = RT\ln(K_d) \quad \text{(S2)}$$

$$\Delta G^\circ \approx -2.303RT \cdot pEC_{50} \quad \text{(S3)}$$

$$\Delta G^\circ \approx -5.71 \cdot pEC_{50}\ \mathrm{kJ/mol} \quad \text{(S4)}$$

However, no significant correlation was observed between raw Vina scores and pEC50 (**Supplementary Fig. S1b**; Spearman's $\varrho = 0.14$, $p = 0.56$), even when allowing one ligand (α-NETA) to be treated as an outlier. This confirmed that raw Vina scores alone are unreliable predictors of activity.

Attempted size correction. We observed a relationship between Vina score and HAC in the full dataset (n = 666). This could not be interpreted solely as Vina bias, as the receptor itself may introduce its own preference for ligands of a particular size. We fitted a smooth single-bend model to the scatter:

$$y = b_0 + b_1 x + b_2 w \ln\left(1 + e^{\frac{x-k}{w}}\right) \quad \text{(S5)}$$

with R^2=0.78 (**Supplementary Fig. S1c**). Based on this, we calculated a "corrected" Vina score:

$$\text{Vina}_{\text{corrected}} = \text{Vina}_{\text{expected}} - \text{Vina}_{\text{observed}} \quad \text{(S6)}$$

However, the corrected scores showed no correlation with experimental pEC50 (**Supplementary Fig. S1d**; Spearman's ϱ = 0.09, p = 0.74). We therefore concluded that direct correction was not effective, likely because the receptor itself is biased toward molecules of a particular size. Interestingly, most known ligands were located in the region of the bend (HAC = 18 ± 5; **Supplementary Fig. S1c**).

Ligand efficiency as the primary metric. We then adopted ligand efficiency (LE) as the primary metric:

$$\text{LE} = \frac{-\text{Vina}_{\text{score}}}{\text{HAC}} \quad \text{(S7)}$$

LE normalises binding energy per heavy atom and has been shown to better reflect activity for GPCR ligands. With the outlier α-NETA excluded, LE showed a trend towards correlation with pEC50 (**Supplementary Fig. S1e**; Spearman's ϱ = 0.43, p = 0.05), which was superior to both raw and corrected Vina scores. We therefore used LE for ranking all docked compounds.

Identification of candidates. Using LE for ranking, we focused on compounds with LE ≥ 0.6 and HAC ≤ 10, i.e. small molecules with efficiency comparable to the known endogenous agonist TMA (LE = 0.596, HAC = 4). Three small amines met these criteria: 2-aminophenol (LE = 0.635), aminoacetone (LE = 0.664), and butan-1-amine (LE = 0.731) (**Supplementary Table S1**). All three correspond to ECs that were significantly altered in TAAR5-KO microbiota (FDR < 0.05). We did not find any previous reports of these compounds being screened against TAAR5.

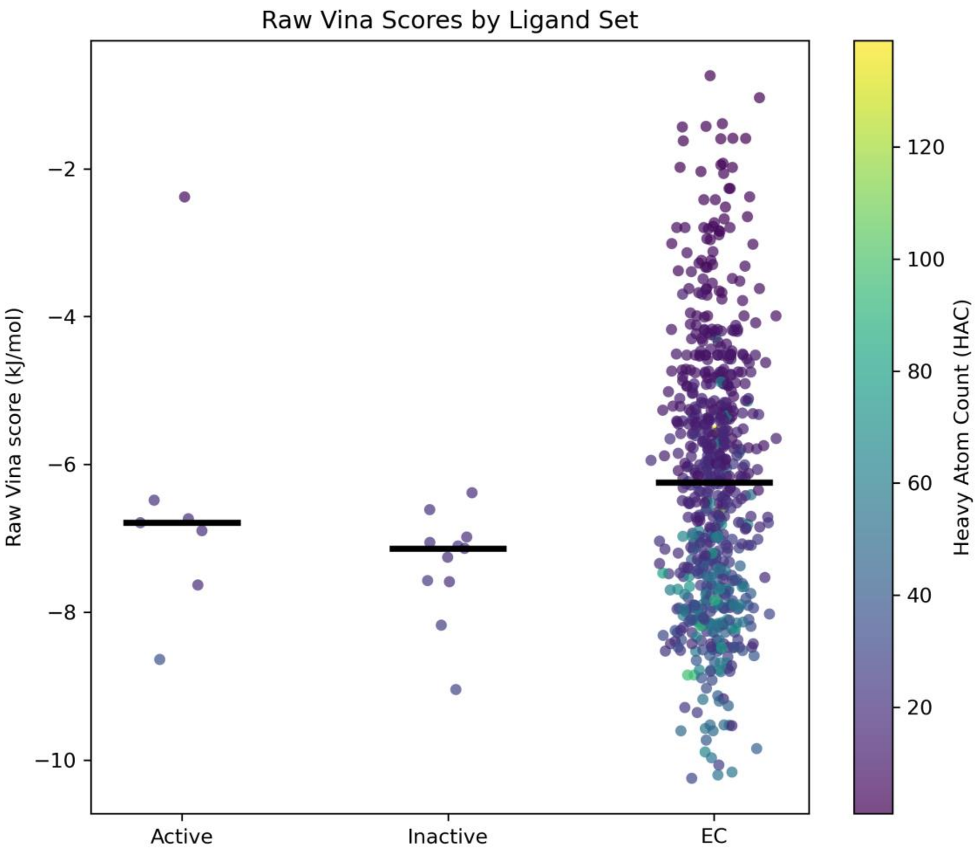
Raw Vina Scores by Ligand Set
Raw Vina score (kJ/mol)
−2
−4
−6
−8
−10
Active
Inactive
EC
Heavy Atom Count (HAC)
120
100
80
60
40
20

(a)

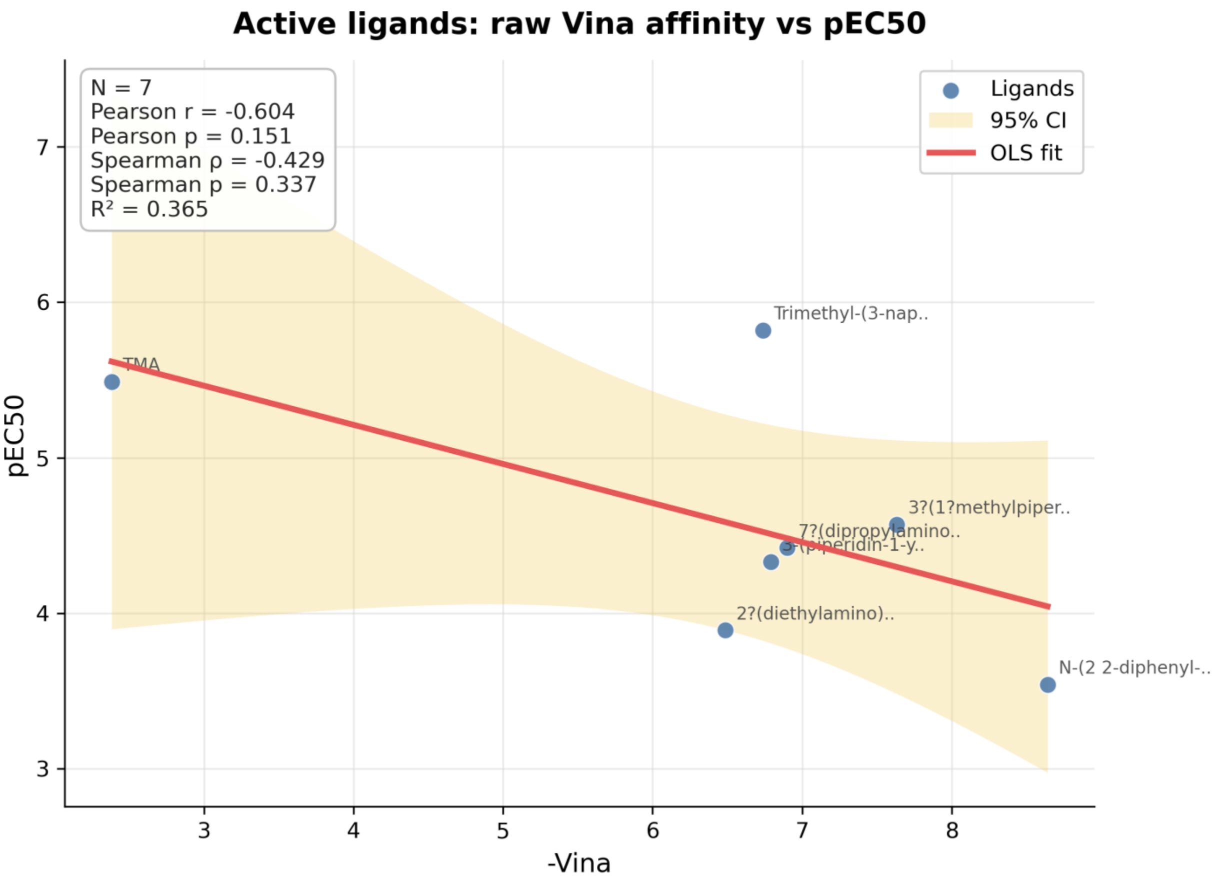
Active ligands: raw Vina affinity vs pEC50
N = 7
Pearson r = -0.604
Pearson p = 0.151
Spearman ρ = -0.429
Spearman p = 0.337
R² = 0.365
Ligands
95% CI
OLS fit
TMA
Trimethyl-(3-nap..
3?(1?methylpiper..
7?(dipropylamino..
3-(piperidin-1-y..
2?(diethylamino)..
N-(2 2-diphenyl-..
pEC50
-Vina
3
4
5
6
7
8

(b)

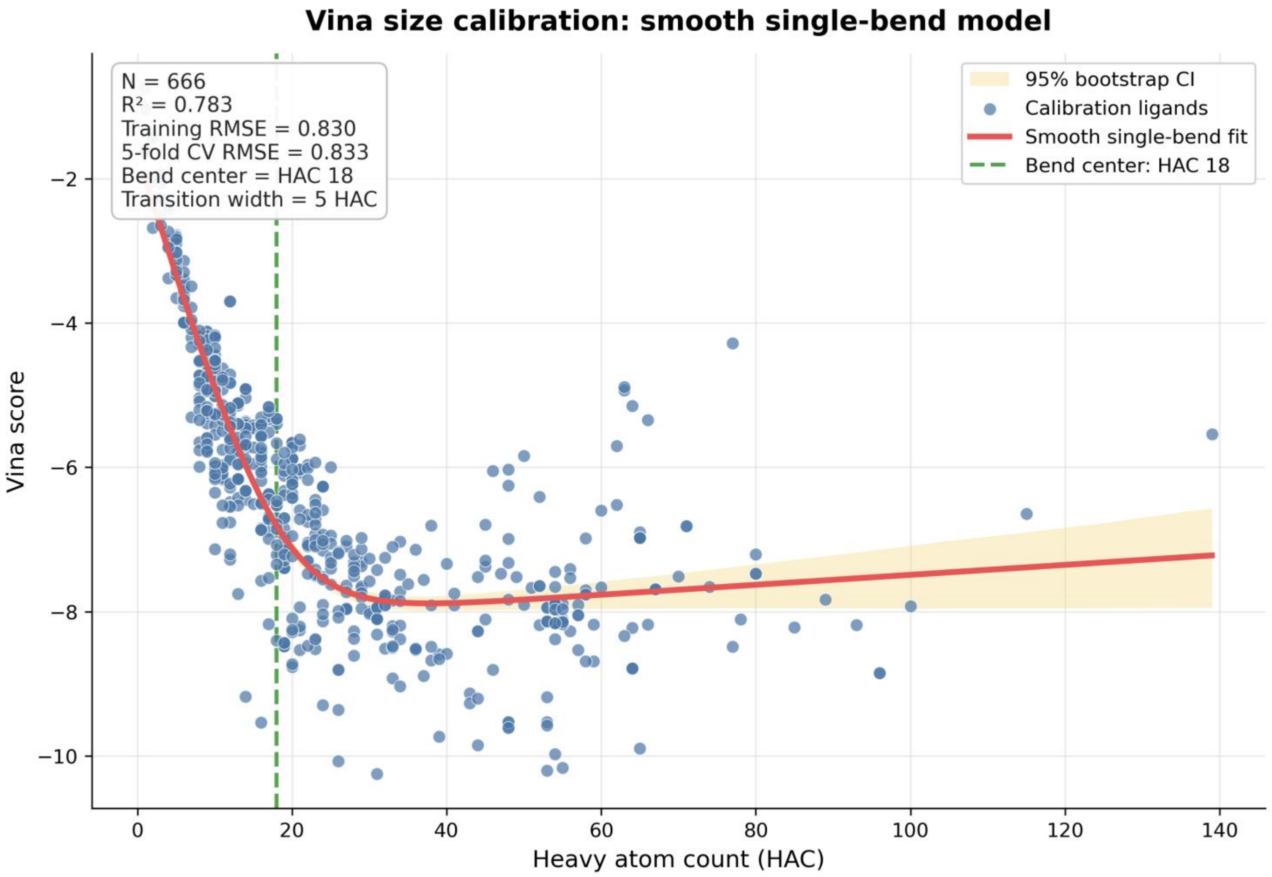
Vina size calibration: smooth single-bend model
N = 666
R² = 0.783
Training RMSE = 0.830
5-fold CV RMSE = 0.833
Bend center = HAC 18
Transition width = 5 HAC
95% bootstrap CI
Calibration ligands
Smooth single-bend fit
Bend center: HAC 18
Vina score
−2
−4
−6
−8
−10
0
20
40
60
80
100
120
140
Heavy atom count (HAC)

(c)

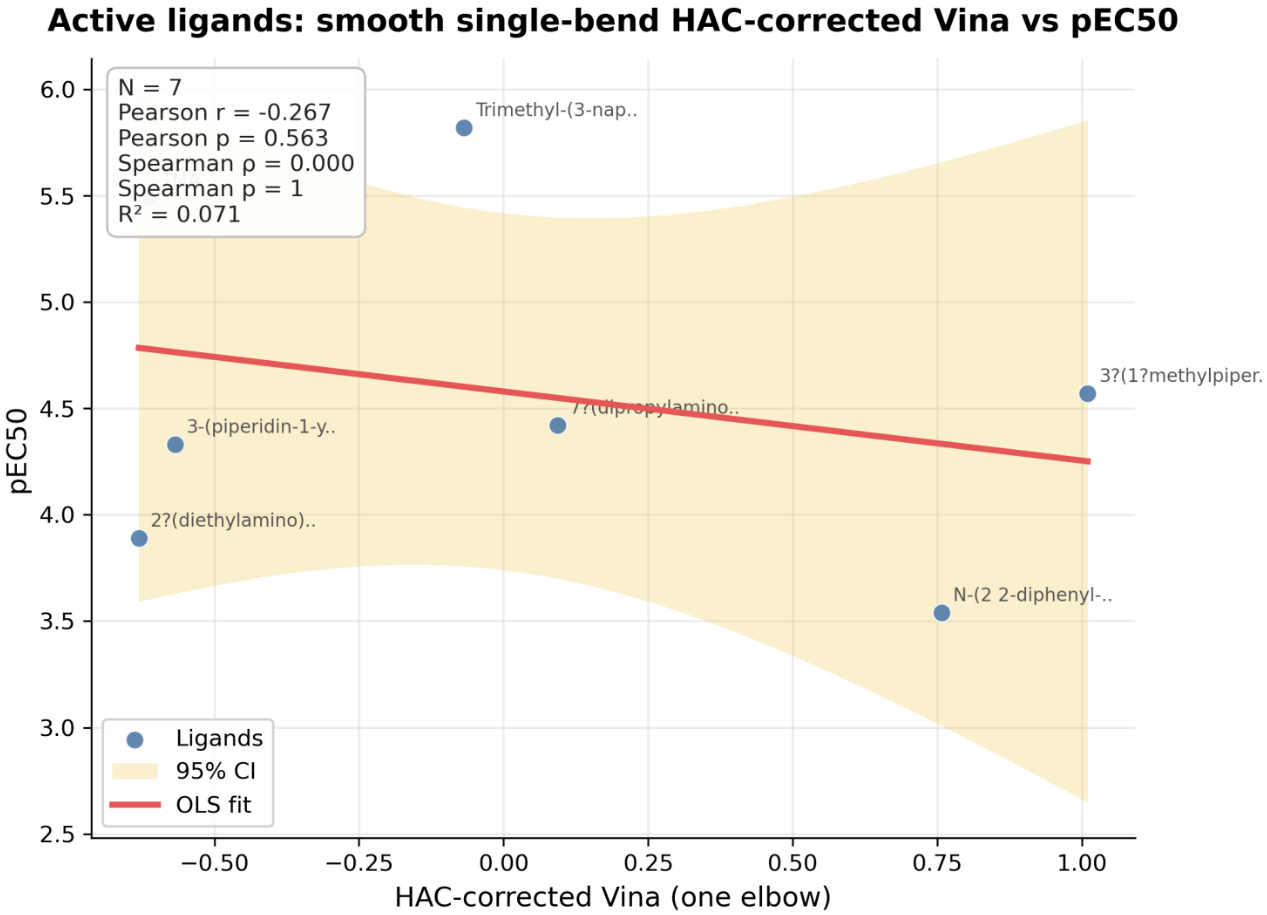
Active ligands: smooth single-bend HAC-corrected Vina vs pEC50
N = 7
Pearson r = -0.267
Pearson p = 0.563
Spearman ρ = 0.000
Spearman p = 1
R² = 0.071
Trimethyl-(3-nap..
3?(1?methylpiper.
7?(dipropylamino..
3-(piperidin-1-y..
2?(diethylamino)..
N-(2 2-diphenyl-..
Ligands
95% CI
OLS fit
pEC50
6.0
5.5
5.0
4.5
4.0
3.5
3.0
2.5
−0.50
−0.25
0.00
0.25
0.50
0.75
1.00
HAC-corrected Vina (one elbow)

(d)

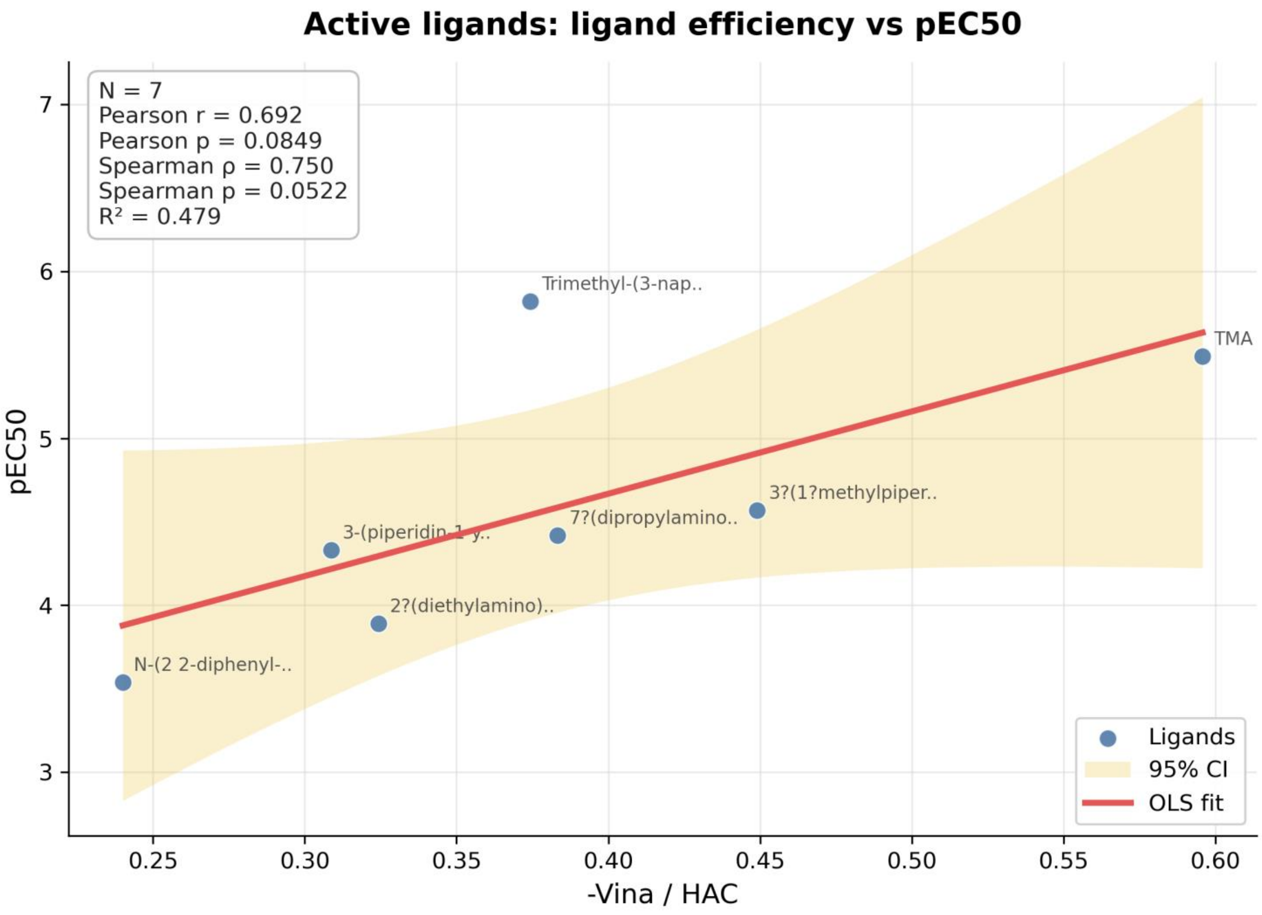

(e)

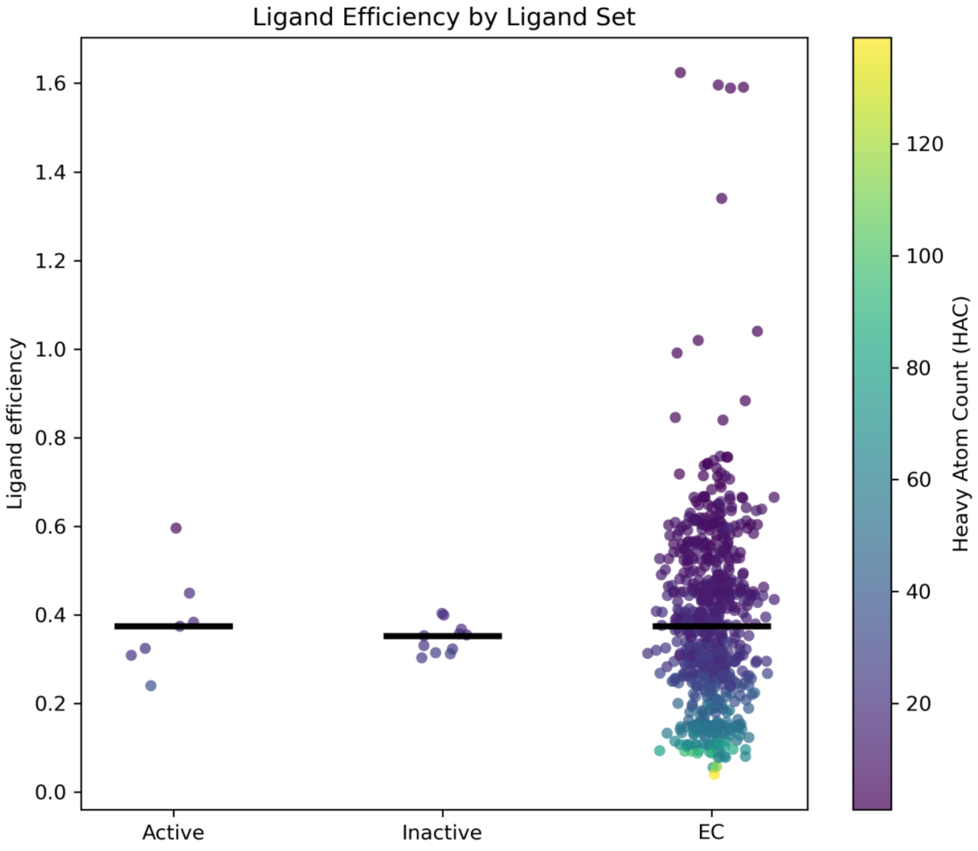

(f)

Supplementary Figure S1. Validation of docking scores and ligand efficiency.

(a) Jitterplot showing raw AutoDock Vina scores vs heavy atom count (HAC) for all 666 docked ligands. Lower Vina scores (more negative) indicate stronger predicted binding. A clear trend towards more favourable scores for larger ligands demonstrates the well-known size bias of the scoring function. **(b)** Raw Vina scores plotted against experimental pEC50 values for known TAAR5 ligands. No significant correlation was observed (Spearman's ϱ = 0.14, p = 0.56), indicating that raw scores are unreliable predictors of activity. **(c)** Smooth single-bend model fitted to the relationship between Vina score and HAC ($R^2 = 0.78$). The model was used to calculate expected Vina scores based on ligand size. **(d)** Corrected Vina scores (Vina_corrected = Vina_expected - Vina_observed) plotted against experimental pEC50. No correlation remained (Spearman's ϱ = 0.09, p = 0.74), indicating that the size bias could not be simply subtracted out. **(e)** Ligand efficiency (LE = -Vina/HAC) plotted against experimental pEC50. With the outlier α-NETA excluded, a trend towards correlation was observed (Spearman's ϱ = 0.43, p = 0.05), validating LE as the primary ranking metric. **(f)** Distribution of ligand efficiency (LE = -Vina/HAC) values for all 666 docked ligands (gray), known TAAR5 agonists (red), and candidate metabolites identified in this study (blue, highlighted). The dashed vertical line indicates the LE of trimethylamine (TMA, LE = 0.596), the reference endogenous agonist. Candidate compounds with LE ≥ 0.6 and HAC ≤ 10 are shown in the inset. This plot illustrates that the identified candidates fall within the region of high ligand efficiency comparable to TMA.

Additional candidates (polycyclic compounds). We also identified several polycyclic compounds with high LE values and sizes comparable to known TAAR5 ligands:

Compound	BRENDA ID	Vina (kJ/mol)	EC	HAC	LE
phenanthrene	4940	-9.174	1.13.11.2	14	0.655
fluoranthene	9536	-9.537	1.13.11.2	16	0.596
9H-carbazole	13154	-7.752	1.13.11.2	13	0.596

All of these substances are linked to catechol 2,3-dioxygenase (EC 1.13.11.2) according to BRENDA. The predicted binding poses of the top candidates are shown in **Supplementary Fig. S2.**

Limitations. All docking results are purely computational and require experimental validation. The homology model, although based on a high-resolution template, has not been experimentally verified. The LE-based ranking provides testable hypotheses, but actual binding and functional activity must be confirmed using in vitro assays (e.g., BRET-based cAMP measurements).

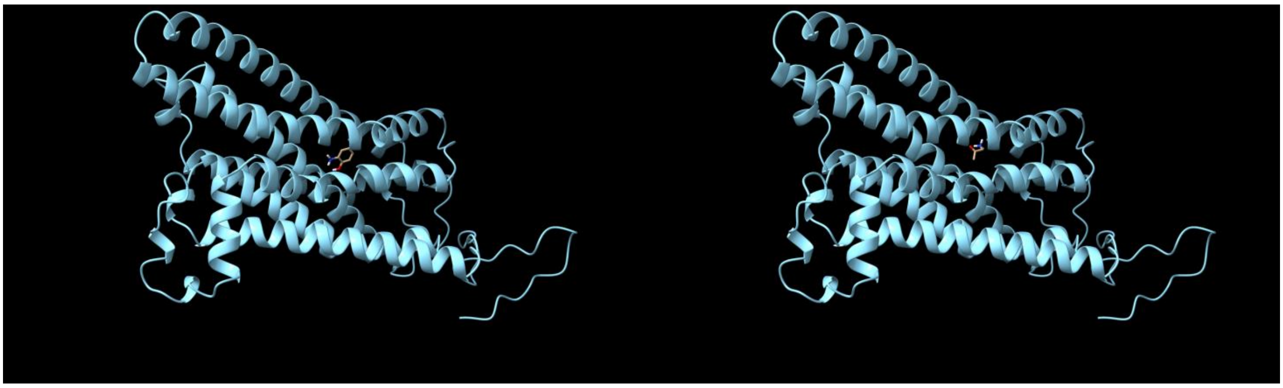

(**a**) (**b**)

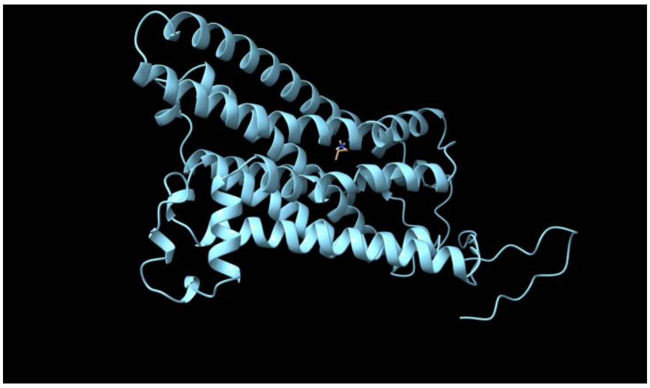

(c)

Supplementary Figure S2. Predicted binding poses of top candidate ligands within the murine TAAR5 homology model **(a)** 2-aminophenol (BRENDA ID: 1910), **(b)** aminoacetone (BRENDA ID: 8045), **(c)** butan-1-amine (BRENDA ID: 1381)

Ligands are shown as sticks (carbon: green, oxygen: red, nitrogen: blue). Key interacting residues are shown as lines. The amine group of each ligand interacts with Asp114 (TM3); additional hydrophobic contacts involve Phe196 and Trp200 (TM5). Docking was performed using AutoDock Vina against a homology model of murine TAAR5 (based on human TAAR1 Cryo-EM structure, PDB: 7XP9).

SUPPLEMENTARY TABLES

Supplementary Table S1. Top candidate microbial metabolites identified by molecular docking

Compound	BRENDA ID	EC number(s)	logFC (KO/WT)	FDR	HAC	Vina (kJ/mol)	LE
2-aminophenol	1910	4.1.1.77, 4.2.1.80	-4.768	0.048	8	-5.081	0.635
aminoacetone	8045	4.1.2.48	+1.026	0.047	5	-3.319	0.664
butan-1-amine	1381	1.4.9.1	-4.747	0.048	5	-3.655	0.731
TMA (reference)	—	—	—	—	4	-2.383	0.596

Shown are compounds with ligand efficiency (LE = -Vina/HAC) ≥ 0.6, comparable to trimethylamine (TMA, LE = 0.596), the known endogenous TAAR5 agonist. EC numbers correspond to enzymes significantly altered in TAAR5-KO microbiota (FDR < 0.05). logFC values indicate KO minus WT; positive values correspond to ECs enriched in KO mice. HAC: heavy atom count; LE: ligand efficiency.

Supplementary Table S2. Polycyclic compounds with ligand efficiency ≥ 0.6

Compound	BRENDA ID	EC	HAC	Vina (kJ/mol)	LE
phenanthrene	4940	1.13.11.2	14	-9.174	0.655
fluoranthene	9536	1.13.11.2	16	-9.537	0.596
9H-carbazole	13154	1.13.11.2	13	-7.752	0.596

These compounds correspond to EC 1.13.11.2 (catechol 2,3-dioxygenase), which was significantly downregulated in TAAR5-KO microbiota (FDR < 0.05). Due to their larger size (HAC 13–16) and hydrophobic nature, they were not prioritized as primary candidates.

Supplementary Table S3. Known TAAR5 ligands used for validation (active controls)

Compound	Abbreviation	HAC	Vina (kJ/mol)	LE	pEC50 (reference)
trimethylamine	TMA	4	-2.383	0.596	6.0
α-NETA	α-NETA	18	-6.737	0.374	5.5
8-OH-DPAT	8-OH-DPAT	18	-6.899	0.383	5.2
BRIL-54443	BRIL-54443	17	-7.633	0.449	5.8
RS 23597	RS 23597	22	-6.791	0.309	5.0
SDZ 205-557	SDZ 205-557	20	-6.486	0.324	4.9

Supplementary Table S4. Inactive compounds used as negative controls

Compound	HAC	Vina (kJ/mol)	LE
ICI 89406	26	-8.177	0.315
N-[2-(piperidinylamino)ethyl]-4-iodobenzamide	18	-7.258	0.403
N-Butyl-N-Ethyl-2-(1-naphthyloxy)ethanamine	20	-7.058	0.353
Ropinirole	19	-6.984	0.368
(+)-UH 232	20	-7.138	0.357
trans-7-Hydroxy-PIPAT	19	-7.589	0.399
Alprenolol	18	-6.385	0.355
S(-)-raclopride	22	-7.105	0.323
Metoclopramide	20	-6.614	0.331
NAN-190	29	-9.048	0.312
WB 4101	25	-7.574	0.303

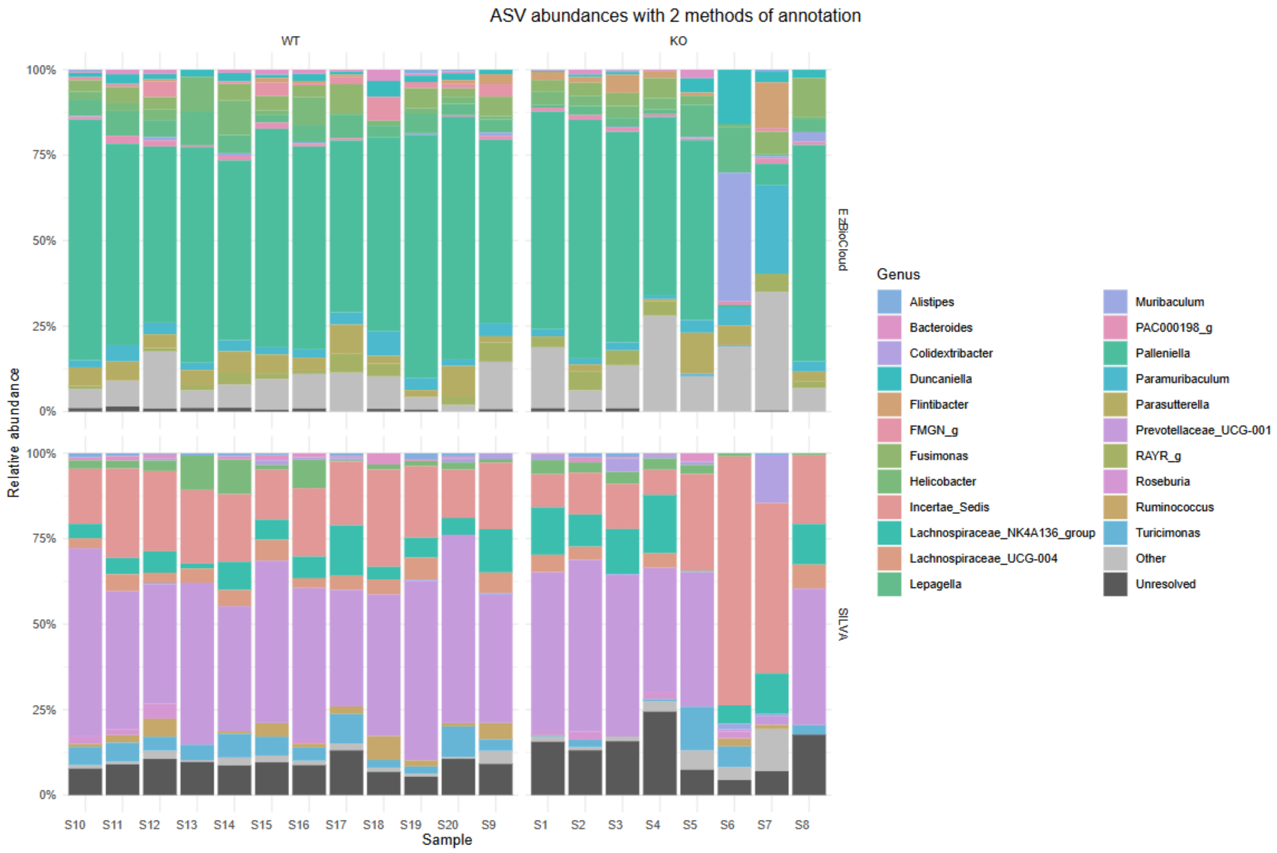

Supplementary Figure S3. Comparison of genus-level taxonomic annotation between SILVA (QIIME2) and EzBioCloud 16S-based ID tool. Relative abundances of ASVs assigned by each method are shown for WT and KO samples. The comparison highlights that while EzBioCloud frequently resolves ambiguous taxa (e.g., assigning Incertae Sedis to Palleniella or Lepagella), SILVA-based annotation tends to remain conservative for incompletely resolved lineages. In our study, we retained SILVA annotations for the main analysis due to its comprehensive reference database for the V3–V4 region and its widespread adoption in 16S rRNA amplicon studies. Taxa with relative abundance < 1% are collapsed into "Other"; ASVs unclassified at the genus level are shown as "Unresolved".

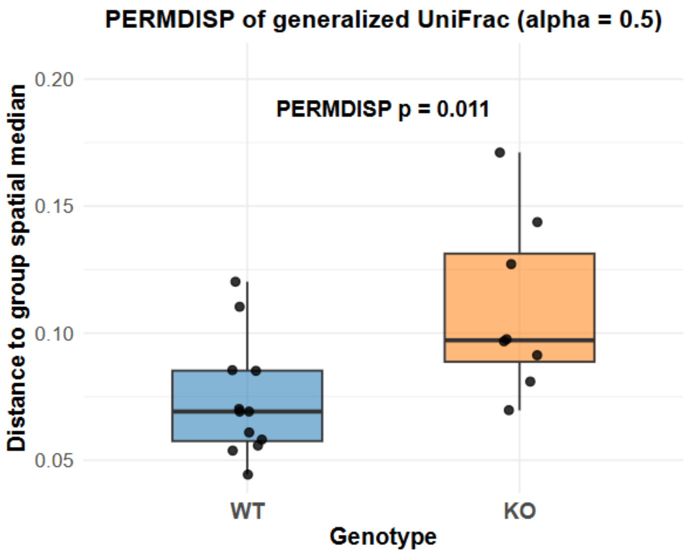

(a)

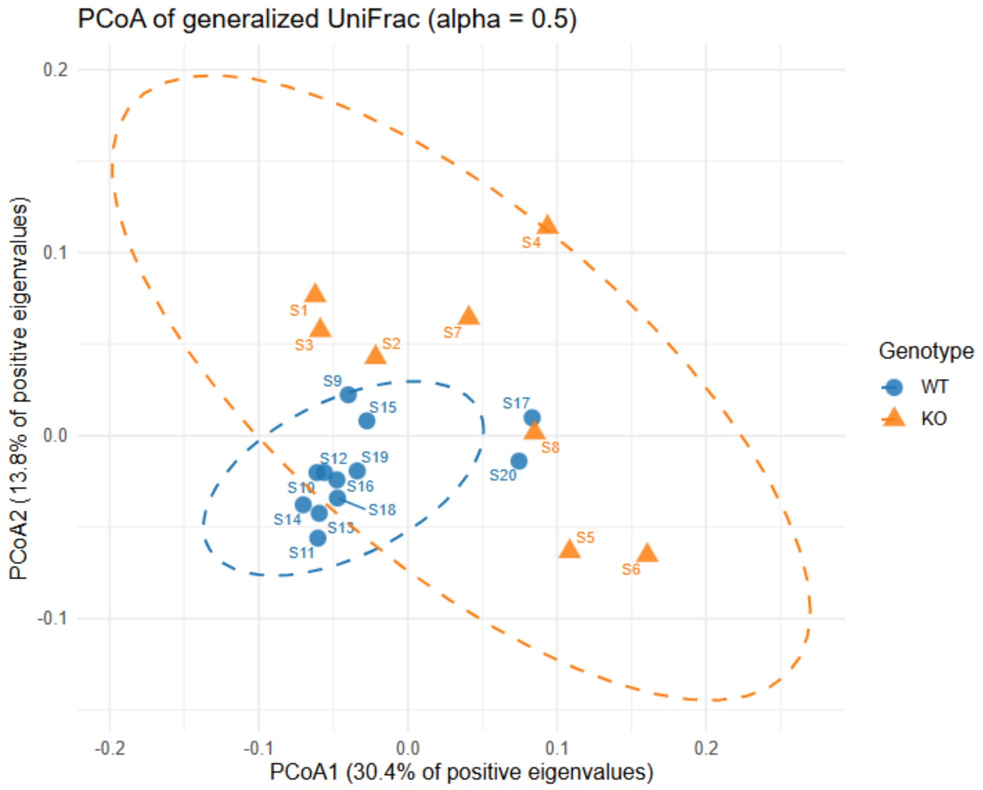

(b)

Supplementary Figure S4. Generalized UniFrac (α = 0.5) beta-diversity analysis of the gut microbiota in TAAR5-KO and WT mice. (a) Boxplot of distances to group centroid (PERMDISP) for WT and KO mice, illustrating multivariate dispersion. PERMANOVA confirmed a significant genotype effect (R^2 = 0.158, F = 3.38, p = 0.0051), and PERMDISP revealed significantly greater dispersion in the KO group (F = 8.03, p = 0.011), (b) Principal coordinate analysis (PCoA) plot based on generalized UniFrac distances. Each point represents an individual sample; WT (blue) and KO (red) groups are shown.
